# Preclinical investigation in FAAH inhibition as a neuroprotective therapy for frontotemporal dementia using TDP-43 transgenic male mice

**DOI:** 10.1186/s12974-023-02792-z

**Published:** 2023-05-06

**Authors:** Irene Santos-García, Carmen Rodríguez-Cueto, Patricia Villegas, Fabiana Piscitelli, Anna Lauritano, Che-Kun J. Shen, Vincenzo Di Marzo, Javier Fernández-Ruiz, Eva de Lago

**Affiliations:** 1 Departamento de Bioquímica y Biología Molecular, Facultad de Medicina, Instituto Universitario de Investigación en Neuroquímica, Universidad Complutense, 28040 Madrid, Spain; 2 Centro de Investigación Biomédica en Red de Enfermedades Neurodegenerativas (CIBERNED), Madrid, Spain; 3 Instituto Ramón y Cajal de Investigación Sanitaria (IRYCIS), Madrid, Spain; 4 Endocannabinoid Research Group, Institute of Biomolecular Chemistry, Consiglio Nazionale Delle Ricerche Pozzuoli, Naples, Italy; 5 The PhD Program for Neural Regenerative Medicine, Taipei Medical University, Taipei, 110 Taiwan; 6 Canada Excellence Research Chair on the Microbiome-Endocannabinoidome Axis in Metabolic Health, CRIUCPQ and INAF, Centre NUTRISS, Faculties of Medicine and Agriculture and Food Sciences, Université Laval, Quebéc City, QC G1V 0A6 Canada

**Keywords:** Frontotemporal dementia, TDP-43, Cannabinoids, Endocannabinoid system, FAAH enzyme, URB597

## Abstract

**Background:**

Frontotemporal dementia (FTD) is a heterogeneous group of early onset and progressive neurodegenerative disorders, characterized by degeneration in the frontal and temporal lobes, which causes deterioration in cognition, personality, social behavior and language. Around 45% of the cases are characterized by the presence of aggregates of the RNA-binding protein TDP-43.

**Methods:**

In this study, we have used a murine model of FTD that overexpresses this protein exclusively in the forebrain (under the control of the CaMKIIα promoter) for several biochemical, histological and pharmacological studies focused on the endocannabinoid system.

**Results:**

These mice exhibited at postnatal day 90 (PND90) important cognitive deficits, signs of emotional impairment and disinhibited social behaviour, which were, in most of cases, maintained during the first year of life of these animals. Motor activity was apparently normal, but FTD mice exhibited higher mortality. Their MRI imaging analysis and their ex-vivo histopathological evaluation proved changes compatible with atrophy (loss of specific groups of pyramidal neurons: Ctip2- and NeuN-positive cells) and inflammatory events (astroglial and microglial reactivities) in both cortical (medial prefrontal cortex) and subcortical (hippocampus) structures at PND90 and also at PND365. The analysis of the endocannabinoid system in these mice proved a decrease in the hydrolysing enzyme FAAH in the prefrontal cortex and the hippocampus, with an increase in the synthesizing enzyme NAPE-PLD only in the hippocampus, responses that were accompanied by modest elevations in anandamide and related *N*-acylethanolamines. The potentiation of these elevated levels of anandamide after the pharmacological inactivation of FAAH with URB597 resulted in a general improvement in behaviour, in particular in cognitive deterioration, associated with the preservation of pyramidal neurons of the medial prefrontal cortex and the CA1 layer of the hippocampus, and with the reduction of gliosis in both structures.

**Conclusions:**

Our data confirmed the potential of elevating the endocannabinoid tone as a therapy against TDP-43-induced neuropathology in FTD, limiting glial reactivity, preserving neuronal integrity and improving cognitive, emotional and social deficits.

**Supplementary Information:**

The online version contains supplementary material available at 10.1186/s12974-023-02792-z.

## Background

Frontotemporal dementia (FTD) is one of the most common causes of dementia after Alzheimer's disease with an incidence of 4.1 cases per 100,000 subjects each year [32]. FTD is a spectrum of different early and progressive disorders characterized by atrophy of the frontal and temporal lobes caused by synaptic and neuronal loss, reactive gliosis, and microvacuolization [33, 58]. As a consequence of this frontotemporal preference, the clinical signs of FTD are related to impairment in cognitive domains (e.g., planning, decision-making, social cognition, language processing), as well as in different executive functions (e.g., working memory, cognitive flexibility, attention, emotional processes, or episodic and semantic memory) [41, 58]. At histopathological and molecular levels, FTD is characterized by the presence of toxic protein aggregates, which are immunologically detectable and generated as a consequence of alterations in protein homeostasis, both in neurons and in glial cells [7]. This allows a classification of FTD based on the main component of these cytoplasmic inclusions: (i) FTLD-Tau (40–45% of genetic cases); (ii) FTLD-TDP-43 (40–45%); (iii) FTLD-FUS (5–10%); and (iv) FTLD-UPS (1%) [49, 58, 68]. This "molecular classification" is, in a certain way, associated with the genetic origin of the disease, as approximately 40–50% of patients present an autosomal dominant inheritance pattern [56], with the remaining 50–60% of cases having sporadic origin [32]. The TDP-43-dependent FTD is one of the most common forms, being also associated with some forms of amyotrophic lateral sclerosis (ALS) [26]. Its most relevant histopathological sign is the presence of positive cytoplasmic inclusions for the nuclear protein TDP-43, which regulates the transcription, stability, transport and processing of RNA [76]. In FTD, these inclusions, in which the TDP-43 protein appears hyperphosphorylated, ubiquitinated and/or partially cleaved, are located mainly in the prefrontal cortex, the dentate gyrus of the hippocampus, and the striatum, leading to intense atrophy of the frontal and temporal lobes accompanied by gliosis and hippocampal sclerosis [76].

Currently, there is no approved neuroprotective treatment for FTD, only multidisciplinary strategies based on the management of different groups of symptoms, together with rehabilitation and speech therapy in those patients with aphasia or dysarthria [43]. An interesting neuroprotective strategy for FTD may be some modulators of the endocannabinoid signaling, a regulatory system largely present in the CNS, which plays key functions in the control of neuronal homeostasis, integrity and survival, and also of other neural cells [22]. Such strategy is being investigated in different neurodegenerative disorders (e.g., Alzheimer’s disease, Parkinson’s disease, Huntington’s chorea, multiple sclerosis, ALS), mainly in preclinical studies with a few clinical trials already finalized or currently in progress [22]. Recent experimental evidence has related Tau-dependent FTD with dysregulation in the endocannabinoid signaling [25], which may support the pharmacological modulation of certain proteins of this system (e.g., CB_2_ receptors) as a promising disease-modifying therapy in this form of FTD [10, 25, 30]. Such potential has not been investigated in TDP-43-dependent FTD yet, so that this has been the major objective of the present study, which has been conducted with a murine conditional TDP-43-dependent model of FTD that overexpress this protein exclusively in the forebrain (cortical and subcortical (e.g. hippocampus, striatum) areas) under the control of α-CaMKII promoter [75]. These authors found that overexpression of TDP-43 in forebrain neurons is enough for these transgenic mice to exhibit numerous behavioural and histopathological abnormalities reminiscent of human FTD associated with the presence of cytosolic aggregates of this protein [75]. For example, they found impaired learning/memory, progressive motor dysfunction, and hippocampal atrophy at 2 and 6 months of age, although the study did not explore older ages and did not separate by sexes [75]. Therefore, our first objective in this study was to confirm that major neuropathological abnormalities characteristics of these mice (using exclusively males) at adult age (postnatal day 90 (PND90) in our study) still persisted at ages (postnatal day 365 (PND365) in our study) older than those investigated by Tsai et al. [75], as well as to identify other behavioural alterations that may also recapitulate additional patient signs (e.g. emotional impairment, altered social behaviour). We also investigated whether these abnormalities provoke a premature death of these mice, as well as whether the medial prefrontal cortex (mPFC) and the hippocampus, which play a key role in the cognitive, social interaction and emotional responses altered in FTD mice, are within the most affected CNS structures in this pathology. These structures were also used to further explore a possible dysregulation in specific elements of the endocannabinoid signaling. Lastly, given that changes found in this system affected the balance between the synthesis and degradation of endocannabinoids, in particular anandamide, changes that could be interpreted as an endogenous protective response, we also explored the therapeutic potential of the inhibition of the key anandamide hydrolysing enzyme FAAH in these mice. To this end, we used the selective inhibitor URB597 [59], which has been also investigated as a potential neuroprotective therapy in different neuronal injury conditions [1, 11, 51, 73, 81, 82].

## Methods

### Animals, experiments and sampling

All animal experiments were conducted with the mouse model of TDP-43-related FTD developed by Tsai et al. [75]. These mice overexpress TDP-43 protein exclusively in the forebrain (under the control of CaMKIIα promoter), which generates elevated levels of TDP-43 in forebrain structures, in particular in the two areas of interest in this study, the mPFC and the hippocampus, with elevations close to 50% (see Table 1). Breedings to generate this colony were generously provided by Dr. Shen (Taipei Medical University, Taiwan) and they were housed in our animal facilities (CAI-Animalario, Faculty of Medicine, Complutense University, ref. ES280790000086) under controlled photoperiod (08:00–20:00 light) and temperature (22 ± 1 °C), and with free access to standard diet and water. All animal experiments were conducted according to local and European rules (directive 2010/63/EU), as well as conformed to ARRIVE guidelines. They were approved by the ethical committees of our university and the regulatory institution (ref. PROEX 059/16).

**Table 1:** Levels of TDP-43 (total and phosphorylated) measured by Western blot (see Additional file 1: Fig. S1 for representative blots) in different forebrain areas of FTD and wildtype male mice at PND90

CNS structures	Marker	Wildtype mice	CamKII-TDP43 mice
mPFC	TDP-43	100.1 ± 5.9 (7)	157.8 ± 14.7 (5)***
	p-TDP-43/TDP-43	100.0 ± 16.6 (6)	159.9 ± 18.6 (5)*
Hippocampus	TDP-43	100.0 ± 4.1 (6)	135.7 ± 12.5 (5)*
	p-TDP-43/TDP-43	100.0 ± 19.4 (6)	129.4 ± 36.7 (5)

Details in the text. Data correspond to percentages over the wildtype group and are expressed as means ± SEM with the number of animals per experimental group in parentheses. They were analysed by the unpaired Student’s t-test (*p < 0.05, **p < 0.01, ***p < 0.005 versus wildtype mice)

In a first experiment, CaMKII-TDP-43 male mice and their wildtype animals were generated from homozigotic breedings. Animals were subjected at PND90 to behavioural testing followed by MRI imaging analysis. Then, animals were euthanized by rapid decapitation. A second cohort of CaMKII-TDP-43 and wildtype mice were left to reach one year of age (PND365), being then subjected to behavioural testing followed by euthanasia as in the above cohort. This second cohort of animals was also used to detect possible motor defects in CaMKII-TDP-43 mice, by recording monthly (from 4 up to 12 months of age) their rotarod performance and the occurrence of clasping response as a marker of dystonia. It was also used to analyze animal survival, using the following criteria to trigger euthanasia: (i) severe weight loss (> 25%); (ii) animals having bristly hair, closed eyes, lethargy or immobility; (iii) paralysis in both hind limbs; and (iv) inability to walk and lack of response to manipulation.

In a second experiment, CaMKII-TDP-43 male mice and their wildtype controls were treated with the selective FAAH inhibitor URB597 (Tocris Bioscience, Bristol, UK) at the dose of 0.2 mg/kg or vehicle (3.3% DMSO + 6% Cremophor in saline solution), both administered i.p. at alternate days, following the procedure described by Piomelli et al. [59]. The treatment was initiated when animals were 45 days old and prolonged each two days up to the age of 89 days, following the data obtained in the first experiment, which indicated 45 days as a presymptomatic age and 90 days as a disease stage with already evident signs of cognitive, emotional and social interaction impairment associated with neuronal losses and neuroinflammatory events. All animals were euthanized by rapid decapitation 24 h after the last injection.

In both experiments, brains were rapidly removed after decapitation. Right hemispheres were fixed for one day at 4 °C in fresh 4% paraformaldehyde prepared in 0.1 M phosphate buffered-saline (PBS), pH 7.4. Samples were cryoprotected by immersion in a 30% sucrose solution for a further day, and finally stored at − 80 °C until to be used for histology (immunofluorescence analysis). Left hemispheres were dissected (mPFC, hippocampus) and frozen by immersion in cold 2-methylbutane followed by storage at − 80 °C until being used for biochemistry (qPCR, Western blot, LC-APCI-MS) analysis.

### Behavioral recording

#### Novel object recognition

The analysis of recognition and working memory was carried out in an opaque methacrylate box (50 × 50 × 50 cm) with a base covered with sawdust, following the procedures described by Antunes and Biala [3] and Lueptow [48]. This procedure allows, among others, the quantification of the discrimination index (time exploring the novel object minus the time exploring the familiar object divided by the total exploration time) and the preference index (time exploring the novel object divided by the total exploration time), in both cases expressed as percentage.

#### T-Maze test

This test was carried out to analyze spatial and short-term working memory using the "spontaneous alternation" paradigm in a T-shaped opaque methacrylate maze (each arm measuring 40 × 10 × 10 cm), according to the procedures described by Hughes [36] and Prieur and Jadavji [62].

#### Water Morris test

The analysis of spatial memory and learning was carried out over four consecutive days in a circular pool (diameter: 120 cm; height: 20 cm) with approximately 9.5 cm of water stained with non-toxic black paint at a temperature of 22 °C, following the procedures described by Morris [55], Vorhees and Williams [79] and López et al. [47]. The data were collected and processed using Smart 3.0 software (Panlab, Barcelona, Spain), which allows to follow the animal trajectory and to quantify the latency time to reach the platform, the swimming speed and the total distance travelled.

#### Elevated Plus Maze

Animal anxiety was measured in the elevated plus maze that consisted of two opposite closed (30 × 5 × 15 cm) and open (30 × 5 cm) arms forming a plus-shaped maze, as described by Walf and Frye [80]. The test allows to quantify the total number of entries into the open and closed arms, and the time spent in the open and closed arms, as well as the risk-taking (number of times the animal approached from the central platform into the open arms without entering them) which may serve as an index of lower impulsivity.

#### Social interaction test

This test was conducted in an open field arena (45 × 45 cm) in which each experimental animal was allowed to freely explore a novel unfamiliar congener for 20 min, using a previously published protocol [70] with some modifications also published [2].

#### Tail suspension test

This test serves to detect animal responses related to apathy and/or anhedonia and was carried out following the procedure described by Steru et al. [72] and Can et al. [9].

#### Spray test

This test was used for the assessment of stereotypic behaviours in mice, more specifically by observing grooming behaviour which can be taken as a pathological sign if developed compulsively (see details in [4, 38]).

#### Rotarod test

Mice were also investigated for possible motor weakness using the rotarod test (LE8200 device; Panlab, Barcelona, Spain) according to the procedure described in Espejo-Porras et al. [19].

#### Clasping response

Hind-limbs clasping behavior was recorded to assess dystonia following a previously published procedure  [78] with some modifications also published [2].

#### Computer-aided actimeter

Motor activity was analyzed in a computer-aided actimeter (Actitrack, Panlab, Barcelona, Spain) as published elsewhere [57] with some modifications also published [2].

### MRI imaging analysis

MRI studies were performed at SIERMAC (Instituto de Investigaciones Biomédicas Alberto Sols, CSIC-UAM, Madrid, Spain) using a Bruker Pharmascan System (Bruker Medical Gmbh, Ettlingen, Germany) according to a procedure previously described (see all methodological details in [20]). For slide quantification, the third ventricle was used as an anatomical marker in both wildtype and CamKII-TDP-43 mice to align, register, and collect images from each animal. The third ventricle was contained in slice 6 on T2-weighted and in slice 3 on apparent diffusion coefficient (ADC)/magnetization transfer (MT) maps, providing a robust anatomical coordinate for the localization of the remaining slices. Three main regions were considered by superimposing the Allen Brain atlas on our MRI slices: whole brain, cerebral cortex, and hippocampus. The data were presented as the ratio of the T2, ADC or MT signal of our region of interest (e.g., cerebral cortex or hippocampus) and the whole brain signal.

### Histological procedures

#### Tissue slicing

Fixed hemibrains were sliced with a cryostat to obtain coronal sections (30 μm thick) that were collected on gelatin-coated slides. Sections were used for procedures of immunofluorescence. For the data presented in the Additional file 8: Fig. S8, spinal cords from FTD and wildtype mice were collected and sliced (L3–L5) for the quantification of spinal motor neurons by using Nissl staining (procedure described in [19]) and for recording glial reactivity by using GPAP and Iba-1 immunofluorescence as indicated below.

### Immunofluorescence

Slices were used for detection and quantification of Ctip-2, NeuN, Sox-2, Ki67, S100-β, GFAP, or Iba-1 immunofluorescence. After preincubation for 1 h with Tris-buffered saline with 0.1% Triton X-100 (pH 7.5), sections were sequentially incubated overnight at 4ºC with the following polyclonal antibodies: (i) anti-Ctip-2 (ref. ab28448, Abcam, Cambridge, UK) used at 1:400; (ii) anti-NeuN (ref. ABN78, Millipore, MA, USA) used at 1:100; (iii) anti-Sox-2 (ref. EPR3131, Abcam, Cambridge, UK) used at 1:200; (iv) anti-S100-β (ref. ab868, Abcam, Cambridge, UK) used at 1:500; (v) anti-Iba-1 (ref. 019-19741, Wako Chemicals, Richmond, VI, USA) used at 1:500; (vi) anti-GFAP (ref. Z0334, Dako Cytomation, Glostrup, Denmark) used at 1:200; or (vii) anti-Ki67 (ref. ab833, Abcam, Cambridge, UK) used at 1:100, followed by washing in Tris-buffered saline and a new incubation (at 37ºC for 2 h) with an anti-rabbit or an anti-mouse, as required, secondary antibody conjugated with Alexa 488 or 546 (Invitrogen, Carlsbad, CA, USA). A DMRB microscope and a DMC4500Fx camera (Leica, Wetzlar, Germany) were used for slide observation and photography.

#### Immunostaining quantification

For quantification of the mean density of immunolabelling or the number of immunostained cells in the selected areas, high-resolution photomicrographs were taken with a 10 × objective under the same conditions of light, brightness and contrast. Counting was carried out with ImageJ software (U.S. National Institutes of Health, Bethesda, Maryland, USA, http://imagej.nih.gov/ij/, 1997–2012). At least 6 images per animal were analyzed to establish the mean of all animals studied in each group. The morphology of immunostained astroglial cells of interest was analyzed using high-resolution digital microphotographs taken with the 40 × objective under the same conditions of light, brightness and contrast. For quantification, we used the protocol described by Young and Morrison [86] to obtain the cytoskeleton of the cells using the FIJI software with the pluggin "Analyze Skeleton 2D/3D", which allow the quantification of the number of branches as well as the cut-off points between them and their length, by evaluating a minimum of 6 cells per animal and 6 animals per experimental group. Each point in the graph represent the mean value of these 6 cells corresponding to an individual mice within each experimental group.

### Real time RT-qPCR analysis

Total RNA was extracted from tissues using Trizol (Life Technologies, Alcobendas, Spain). The total amount of RNA extracted was quantified by spectrometry at 260 nm and its purity was calculated as the ratio between the absorbance values at 260 and 280 nm. RNA integrity was confirmed in agarose gels. DNA was removed and single-stranded complementary DNA was synthesized from 0.5 μg of total RNA using a commercial kit (Rneasy Mini Quantitect Reverse Transcription, Qiagen, Izasa, Madrid, Spain). The reaction mixture was kept frozen at – 20 ºC until enzymatic amplification. Quantitative real-time PCR assays were performed using TaqMan Gene Expression Assays (Applied Biosystems, Foster City, CA, USA) to quantify mRNA levels for TNF-α (Mm99999068_m1), IL-1β (Mm00434228_m1), EAAT2 (Mm012758_m1), Arg-1 (Mm00475988_m1), CB_1_ receptor (Mm00432621_s1), CB_2_ receptor (Mm00438286_m1), FAAH (Mm00515684_m1), MAGL (Mm00449274_m1), DAGL (Mm00813830_m1) and NAPE-PLD (Mm00724596_m1), using GAPDH expression (Mm99999915_g1) as an endogenous control gene for normalization. The PCR assay was performed using the StepOne Plus Real Time PCR System (Applied Biosystems, Foster City, CA, USA) and the threshold cycle (Ct) was calculated by the instrument’s software (Sequence Detection, Applied Biosystems, Foster City, CA, USA). Expression levels were calculated using the 2^−ΔΔCt^ method, but, for presentation, data were transformed to the % over the mean obtained in the wild-type group for each parameter.

### Western blot

Frozen tissues were homogenized in an ice-cold radioimmunoprecipitation assay (RIPA) buffer for protein extraction. Homogenates were centrifuged at 10,000 × *g* for 15 min at 4 °C. Bio-Rad DC protein assay kit (Bio-Rad Laboratories, CA, USA) was used to quantify protein concentration, using bovine serum albumin (BSA) as the standard protein. Then, 15 μg of protein were boiled for 5 min in Laemmli SDS loading buffer (10% glycerol, 5% SDS, 5% β-mercaptoethanol, 0.01% bromophenol blue, and 125 mM TRIS–HCl, pH 6.8) and loaded onto a 12% acrylamide gel (TGX Stain-free Gel FastCast; Bio-Rad Laboratories, CA, USA). After electrophoresis, proteins were transferred to PVDF membranes (Immobilon-P, Millipore, MA, USA) using mini Trans-Blot Electrophoretic transfer cell (Bio-Rad Laboratories, CA, USA). Membranes were then blocked for 1 h at room temperature with Tris-buffered saline containing 5% nonfat dried milk and 0.1% Tween-20 and incubated overnight at 4 °C with the following primary polyclonal antibodies: (i) anti-FAAH (CAY-101600, Cayman Chemicals, MI, USA) used at 1/1000; (ii) anti-CB_1_ (CB1-Rb-Af380, Frontier Institute, Hokkaido, Japan) used at 1/500; (iii) anti-TDP-43 (ref. 10,782–2-AP, Proteintech, Manchester, UK) used at 1:1000; (iv) anti-pTDP43 (ref. 10,782–2-AP, Proteintech, Manchester, UK) used at 1:500; (v) anti-cleaved-caspase 3 (Asp175) (ref. 9664; Cell Signaling, MA, USA) used at 1:100; (vi) anti-ubiquitin (ref. Z0458, Dako, CA, USA) used at 1:1000; (vii) anti-p62 (ref. 610,833, Bd Biosciences, Madrid, Spain) used at 1:1000; or (viii) anti-LC3 (ref. L8918, Sigma, CA, USA) used at 1:1000. Membranes were finally incubated with an ECL horseradish peroxidase-linked whole secondary antibody (GE Healthcare UK Limited, Buckinghamshire, UK) used at a 1:5000 dilution for 2 h at room temperature. Reactive bands were detected by chemiluminescence with the Amersham ECL Prime Western Blotting Detection Reagent (GE Healthcare UK Limited, Buckinghamshire, UK). Images were analyzed with Image Lab software (Bio-Rad Laboratories, CA, USA). Data were calculated as the ratio between the optical densities of the specific protein band and the total protein measured in membranes, and then normalized as percentages over the values of wild-type mice.

### Analysis of endocannabinoid levels

Tissues were homogenized with chloroform/methanol/Tris–HCl 50 mM (2:1:1) containing 5 pmol of d^8^-anandamide, and 50 pmol of d^4^-palmitoylethanolamide (PEA), d^24^-oleylethanolamide (OEA) and d^5^-2-AG (Cayman Chemicals, MI, USA). Homogenates were centrifuged at 10,000 rpm for 1 min (4 ºC), the aqueous phase plus debris were collected and extracted again four times with 1 ml of chloroform. The organic phases from the four extractions were pooled and the organic solvents evaporated under nitrogen. Lyophilized extracts were resuspended in chloroform/methanol 99:1 by vol. The solutions were then purified by open bed chromatography on silica as described [6]. Fractions eluted with chloroform/methanol 9:1 by vol. (containing anandamide, 2-AG, OEA and PEA) were collected and the excess solvent evaporated with a rotating evaporator, and aliquots analyzed by isotope dilution-liquid chromatography/atmospheric pressure chemical ionisation/mass spectrometry (LC-APCI–MS) carried out under conditions described previously [87] and allowing the separations of 2-AG, anandamide, OEA and PEA. MS detection was carried out in the selected ion monitoring mode using m/z values of 356 and 348 (molecular ion + 1 for deuterated and undeuterated anandamide), 384.35 and 379.35 (molecular ion + 1 for deuterated and undeuterated 2-AG), 304 and 300 (molecular ion + 1 for deuterated and undeuterated PEA), and 330 and 326 (molecular ion + 1 for deuterated and undeuterated OEA). The amounts of endocannabinoids and related *N*-acylethanolamines were expressed as pmol/mg of lipid extract.

### Statistics

Data were assessed using, as required, Student’s t-test (for comparison of only two groups), and one-way or two-way ANOVA followed by the Bonferroni test (in cases of multiple comparison required), using GraphPad Prism, version 8.00 for Windows (GraphPad Software, San Diego, CA, USA). Survival data were assessed using Log-Rank test and presented with a Kaplan–Meier analysis, whereas imaging data were analyzed against NOR data in the same CaMKII-TDP-43 mice using linear regression analysis to obtain the Pearson’s correlation coefficient. A p value lower than 0.05 was used as the limit for statistical significance. The sample sizes in the different experimental groups were always ≥ 5, except in a few cases (indicated in legends or visible in scatter plots) due to elimination of some outliers that accomplished the statistical requirements (difference versus mean > 3xSEM) for being not considered.

## Results

### Neuropathological characterization of FTD mice: behavioural data

This study has been conducted in a murine model of FTD that overexpress TDP-43 protein exclusively in the forebrain using the control of CaMKIIα promoter, which concentrates the damage predominantly in cortical and subcortical (hippocampus) neurons [75]. Our first objective was to confirm the TDP-43 overexpression in the two CNS structures of interest in experimental FTD (mPFC and hippocampus). Our data presented in Table 1 indicated higher levels of TDP-43 in CaMKII-TDP-43 mice compared to wildtype animals at PND90 in these two structures (30–50% higher), data that were equivalent to those reported by Tsai et al. [75]. We also measured the ratio between phosphorylated and total TDP-43, which may serve as an index to detect possible formation of aggregates in which p-TDP-43 is abundant, and we found higher values in FTD mice compared to wildtype animals in the mPFC, but this did not happen in the hippocampus (data in Table 1 and blots in Additional file 1: Fig. S1).

**Fig. 1:**
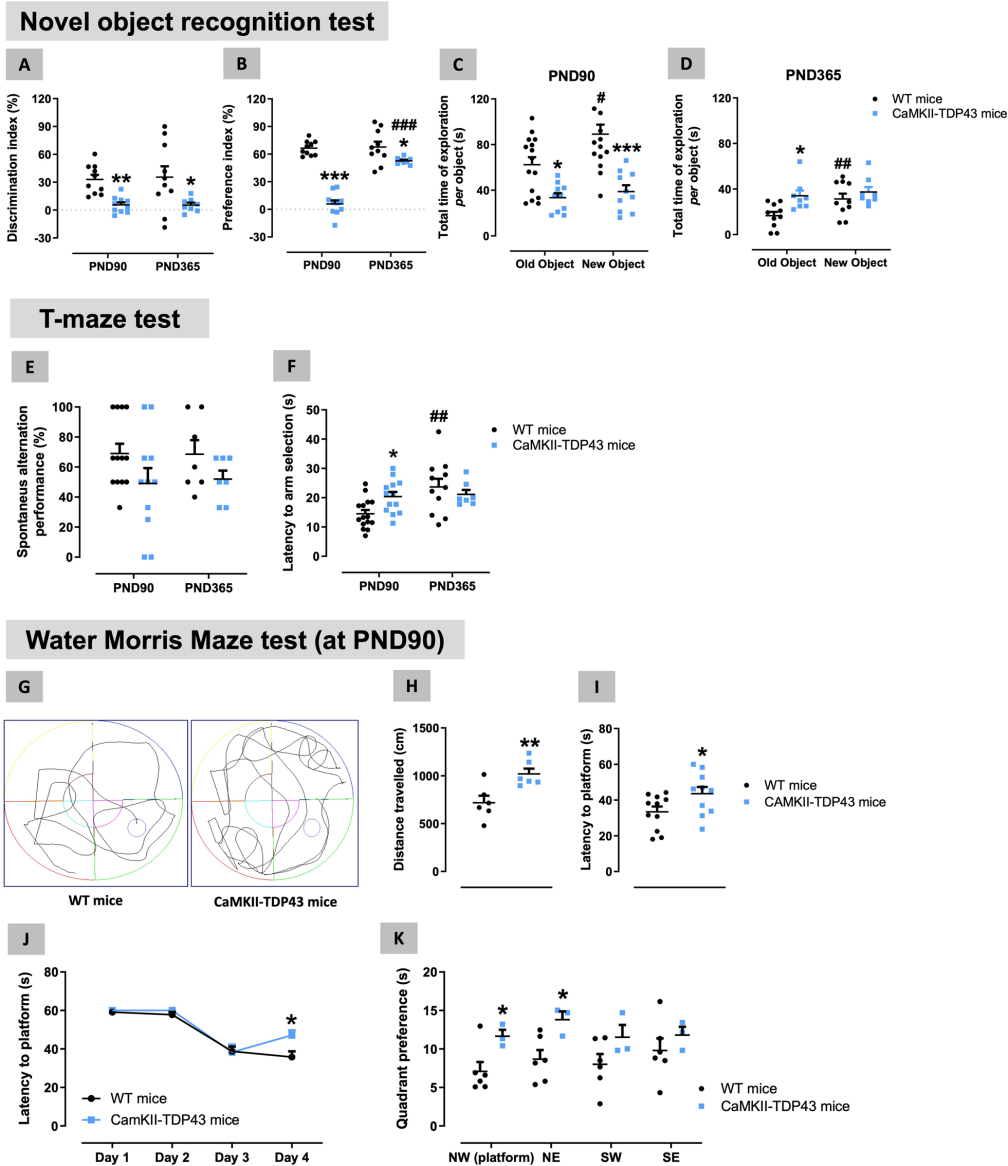
Behavioural data obtained in FTD and wildtype male mice at two different ages (PND90 and PND365) in the Novel Object Recognition (panels **A**–**D**), T-Maze (panels **E**,**F**) and Water Morris Maze (panels **G**–**K**; data correspond only to PND90 with one representative recording for each experimental group included) tests. Details in the text. Data were expressed as means ± SEM and were analysed by two-way analysis of variance (with repeated measures in some cases) followed by the Bonferroni test (*p < 0.05, **p < 0.01, ***p < 0.005 versus the corresponding wildtype mice; #p < 0.05, ##p < 0.01, ###p < 0.005 versus the same genotype at PND90)

With this overexpression, mice at PND90 (1 month older than [75]) exhibited a pathological phenotype characterized by numerous behavioural abnormalities that resulted to be reminiscent first of cognitive (working, recognition and spatial memory) changes seen in FTD patients. For example, they performed worse in the NOR test, as also described in Tsai et al. [75], showing times of exploration which were similar for each object, but significantly lower compared to wildtype animals, which explored more time the new object (genotype: F(1,23) = 29.5, p < 0.0001; Fig. 1A–C). This results in an important reduction in the discrimination (genotype: F(1,18) = 15.6, p < 0.001) and preference (genotype: F(1,18) = 83.9, p < 0.0001) indices in FTD mice compared to wildtype animals (Fig. 1A,B). These differences were not associated with any spatial preference, as they were not visible in the training phase (genotype: F(1,16) = 1.30, ns; genotype x object position: F(1,16) = 0.37, ns; see Additional file 2: Fig. S2). Similar cognitive deficiencies were evident in the T-maze test, as FTD mice at PND90 spent more time (higher latency) to select one of the arms (genotype x age: F(1,42) = 4.60, p < 0.05; Fig. 1F) and proved a trend towards a reduction in the % of spontaneous alternance (genotype: F(1,34) = 4.33, p < 0.05; Fig. 1E) compared to the responses of wildtype mice. Such cognitive deterioration was also visible in the Water Morris Maze test (Fig. 1G–K; similar to [75], at 2 months of age), in which FTD mice at PND90 showed an elevated latency to find the platform (in particular, at the last day of analysis: genotype x day: F(3,51) = 3.18, p < 0.05, Fig. 1I,J) associated with a longer distance travelled (Fig. 1H), as well as with a greater preference for certain quadrants (e.g., the one where the platform is located; see Fig. 1G) (genotype: F(1,21) = 6.24, p < 0.05; Fig. 1K), all in comparison with the performance exhibited by wildtype animals. No changes were seen in the swim speed (data not shown), then reflecting no motor deficiencies (see below).

**Fig. 2:**
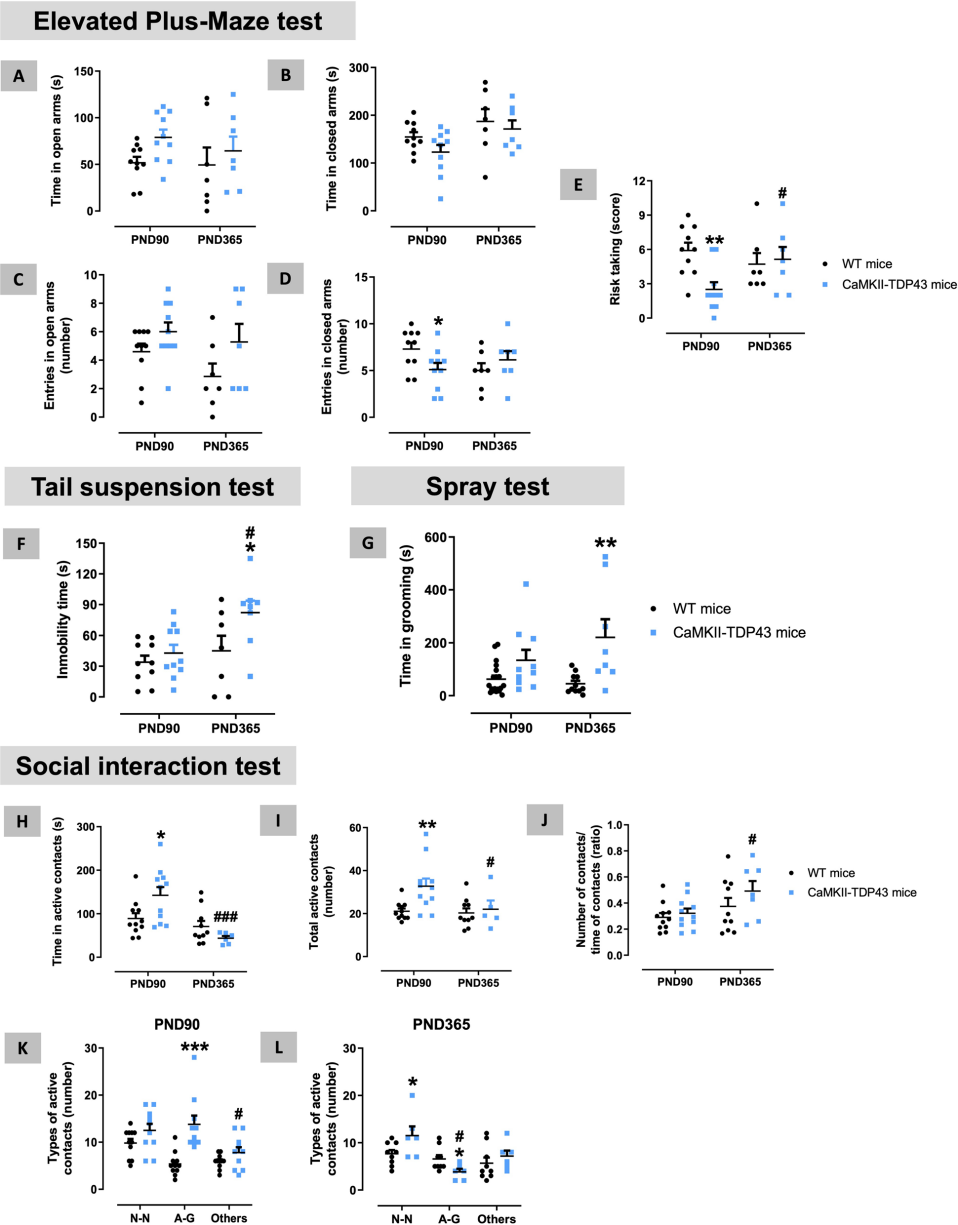
Behavioural data obtained in FTD and wildtype male mice at two different ages (PND90 and PND365) in the Elevated Plus Maze (panels **A**–**E**), Tail Suspension (panel **F**), Spray (panel **G**), and Social Interaction (panels **H**–**L**) tests. Details in the text. Data were expressed as means ± SEM and were analysed by two-way analysis of variance (with repeated measures in some cases) followed by the Bonferroni test (*p < 0.05, **p < 0.01, ***p < 0.005 versus the corresponding wildtype mice; #p < 0.05, ###p < 0.005 versus the same genotype at PND90). *N–N*  nose-nose, *A-G*  anogenital

Our FTD mice also showed impairment in mood-like signs at PND90 measured in the elevated plus maze test (Fig. 2A–E). Thus, we found trends towards an elevation in the time in open arms (Fig. 2A), and the opposite in the closed arms (Fig. 2B), although these changes did not reach statistical significance. However, such statistical significance did reach in the number of entries in open (genotype: F(1,18) = 4.859, p < 0.05; Fig. 2C) and closed (genotype x age: F(1,12) = 5.627, p < 0.05; Fig. 2D) arms, despite the post-hoc test left part of these differences as mere trends (Fig. 2C,D). An additional interesting parameter derived from the elevated plus maze test was the risk taking (to explore an arm without leaving the other arm), which was significantly reduced in FTD mice at PND90 (genotype x age: F(1,12) = 8.572, p < 0.05; Fig. 2E), then suggesting a possible greater impulsivity response (or a reduced level of anxiety). FTD mice also proved a higher (trend at PND90) time spent in grooming in comparison with wildtype mice (genotype: F(1,27) = 15.07, p < 0.005; Fig. 2G) in the spray test, which allows the measure of this stereotyped behaviour that is repeated in a compulsive manner after water nebulization in the mouse vibrissae. FTD mice also exhibited a disinhibited social behaviour (more time spent in interacting with their partners) measured in a social interaction test at PND90 (Fig. 2H–L), reflected in elevated time spent in (genotype x age: F(1,14) = 8.04, p < 0.05; Fig. 2H) and number (genotype: F(1,33) = 5.289, p < 0.05; Fig. 2I) of active contacts. These were, in general, anogenital contacts (genotype: F(1,19) = 12.37, p < 0.005; Fig. 2K), whereas social contacts (nose-nose) were not altered at PND90 (Fig. 2K), although this changed at longer times (see below). These results recapitulated the responses found in patients, which frequently show signs of sexual disinhibition at the onset of the disease but not later [64]. Such altered social interaction behaviour is also concordant with the impulsivity detected in the elevated plus-maze test.

As already indicated before, most of these behavioural abnormalities were seen at PND90, but we were also interested in confirming whether they are maintained during the first year of life of these animals (up to PND365), a fact not investigated in Tsai et al. [75]. Thus, we found, in general, a similar performance by FTD mice in the T-maze test (Fig. 1E) and, in particular, the NOR test (Fig. 1A–D) at PND365, although some of the impaired parameters exhibited certain attenuation (e.g. preference index in the NOR test (Fig. 1B), latency to arm selection in the T-maze (Fig. 1F)). In some cases, these attenuations may be caused by a reduced response, also evident in wildtype mice (e.g., reduction in the time of exploration in the NOR test; Fig. 1D), which may reflect a possible effect of physiological aging. Unfortunately, we could not obtain reliable data in the Water Morris Maze test in mice at PND365, as this test resulted to be too aggressive for animals (both FTD and wildtype) already showing some signs of behavioural deterioration that affected their response in this test.

Attenuations similar to those found in the NOR and T-maze test at PND365 also happened with the impaired response of FTD mice in the elevated plus-maze test at PND90, which was practically normalized at PND365 (Fig. 2A–E). However, other responses were maintained and even intensified at PND365 (e.g., spray test; Fig. 2G), or were visible only at this age but not at PND90, for example a greater immobility in the tail suspension test (F(1,18) = 5.276, p < 0.05; Fig. 2F), which may reflect apathy, anhedonia or depressive state. As regards to the response of FTD mice at PND365 in the social interaction test, our data also indicated a clear attenuation, with similar number and time spent in active contacts compared with wildtype mice (Fig. 2H–J). In addition, the profile of active contacts in FTD mice versus wildtype animals was different at PND365 compared with PND90, showing elevated nose-nose contacts, meaning more social contacts (“sociability”), but reduced anogenital interactions (Fig. 2K,L), as well having, in general, less durable contacts at PND365 (Fig. 2K,L).

**Fig. 3:**
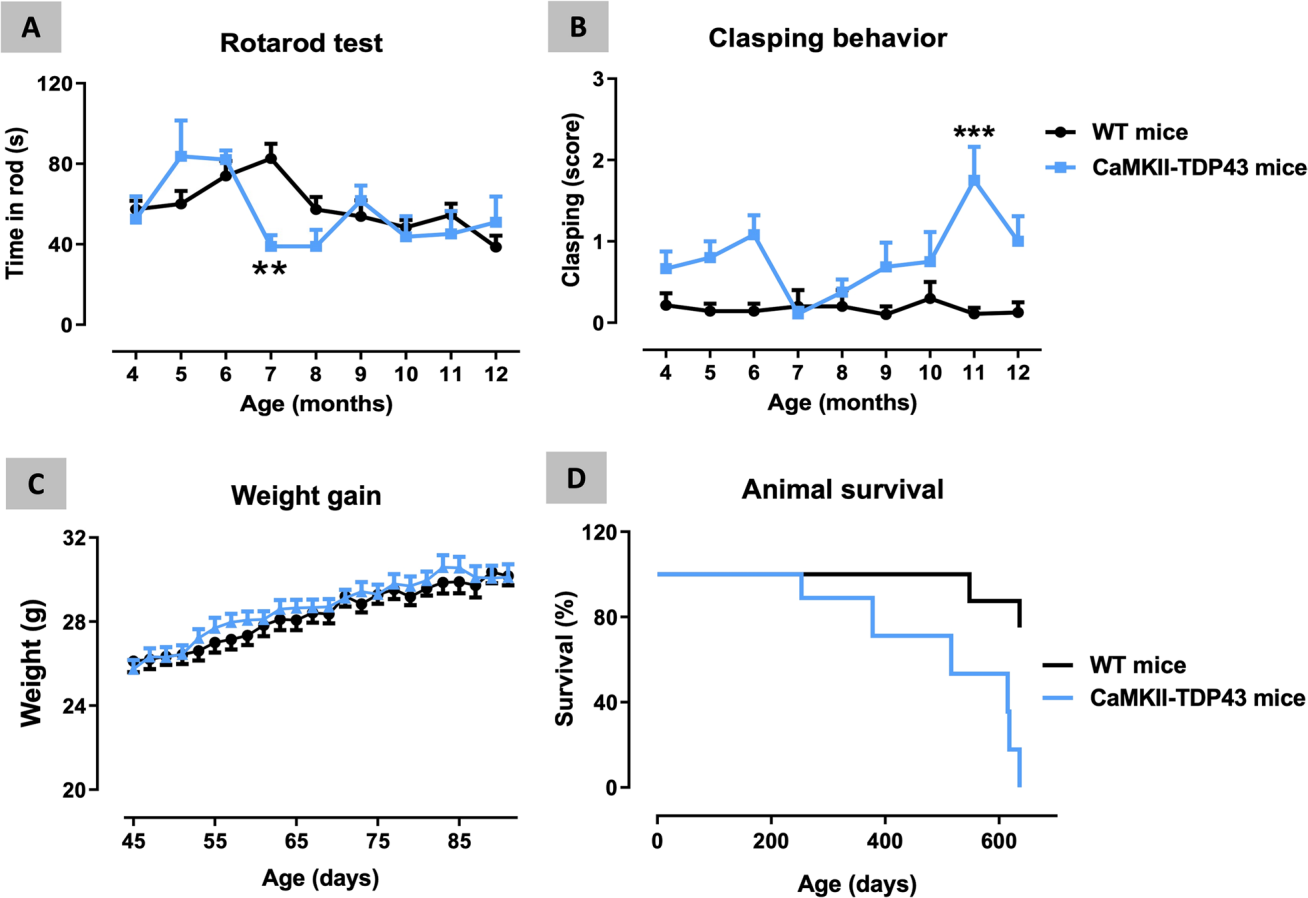
Behavioural data obtained in FTD and wildtype male mice at different ages (from 4 up to 12 months of age) in the Rotarod (panel **A**) and Clasping behaviour (panel **B**) tests, as well as data of weight gain (from 45 up to 90 days of age) (panel **C**) and animal mortality (panel **D**) presented in a Kaplan–Meier plot. Details in the text. Data were expressed as means ± SEM of 8 wildtype mice and 7 FTD animals for motor parameters and weigth gain, and 11 wildtype mice and 9 FTD animals for animal survival. Data were analysed by two-way analysis of variance with repeated measures followed by the Bonferroni test (**p < 0.01, ***p < 0.005 versus the corresponding wildtype mice), except for animal survival that were assessed by Long-Rank test (or Chi-square test)

As regards to the motor behaviour, FTD and wildtype mice were monthly analysed in the rotarod test at ages from 4 up to 12 months (Fig. 3A). Our data indicated no changes in most of ages analysed (only a transient reduction was visible at 7 months of age; Fig. 3A) supporting that motor coordination was not, in general, affected in FTD mice, which is concordant with the lack of TDP-43 overexpression in the cerebellum [75]. Clasping behaviour was also analysed at the same age range (Fig. 3B), proving a certain trend towards to be elevated at several ages, but reaching statistical significance only at 11 months of age (genotype x age: F(8,85) = 3.044, p < 0.01). Such effect may reflect the occurrence of some initial signs of dystonia in FTD mice compared to wildtype animals, although it is important to remark that the response was, in general, very modest with values in FTD mice around 1, reaching a maximum of 2 (only at 11 months of age) in a scale up to 4 (Fig. 3B). Lastly, animals were analysed in the computer-aided actimeter just before to be euthanized at PND90 or PND365 (Additional file 3: Fig. S3), but no changes were detected in any of the different parameters (e.g., ambulation, resting time, velocity, frequency of fast or slow movements) at the two ages analyzed in this test. This lack of relevant and persistent changes in motor behaviour contrasts with the results published by Tsai et al. [75], who did find some motor anomalies from ages older than 6 months, although not associated with alteration in the spinal cord (see below). Such disparity may be related to the fact that experimental groups in the original work by Tsai et al. [75] were formed by males and females together in equivalent proportion, whereas our cohorts were only formed by males. It might be possible that motor alterations are visible only in females, a fact that would be reflected in the data by Tsai et al. [75], but not in our study, although such possibility would require additional research pending to be carried out.

### Neuropathological characterization of FTD mice: weight gain, animal survival and in vivo imaging

Weight gain was also daily measured in FTD and wildtype animals from PND45 up to PND90, the first timepoint used for behavioural and histopathological analysis, but no changes were evident during this period (Fig. 3C). No further analyses were carried out for this parameter. We also recorded the animal survival and found an accelerated mortality in FTD mice, which already appears around 250 days (8–9 months) of age, progressing up to have all animals died before two years of age (Fig. 3D). Mortality in the wildtype group was significantly smaller with only 25% of animals died in the same age interval and the first death appearing approximately at one year and half. The median for FTD mice was 615 and the differences compared with wildtype animals were statistically significant (χ^2^ = 9.723, p < 0.005; Fig. 3D). These results are mostly similar to Tsai et al. [75].

**Fig. 4:**
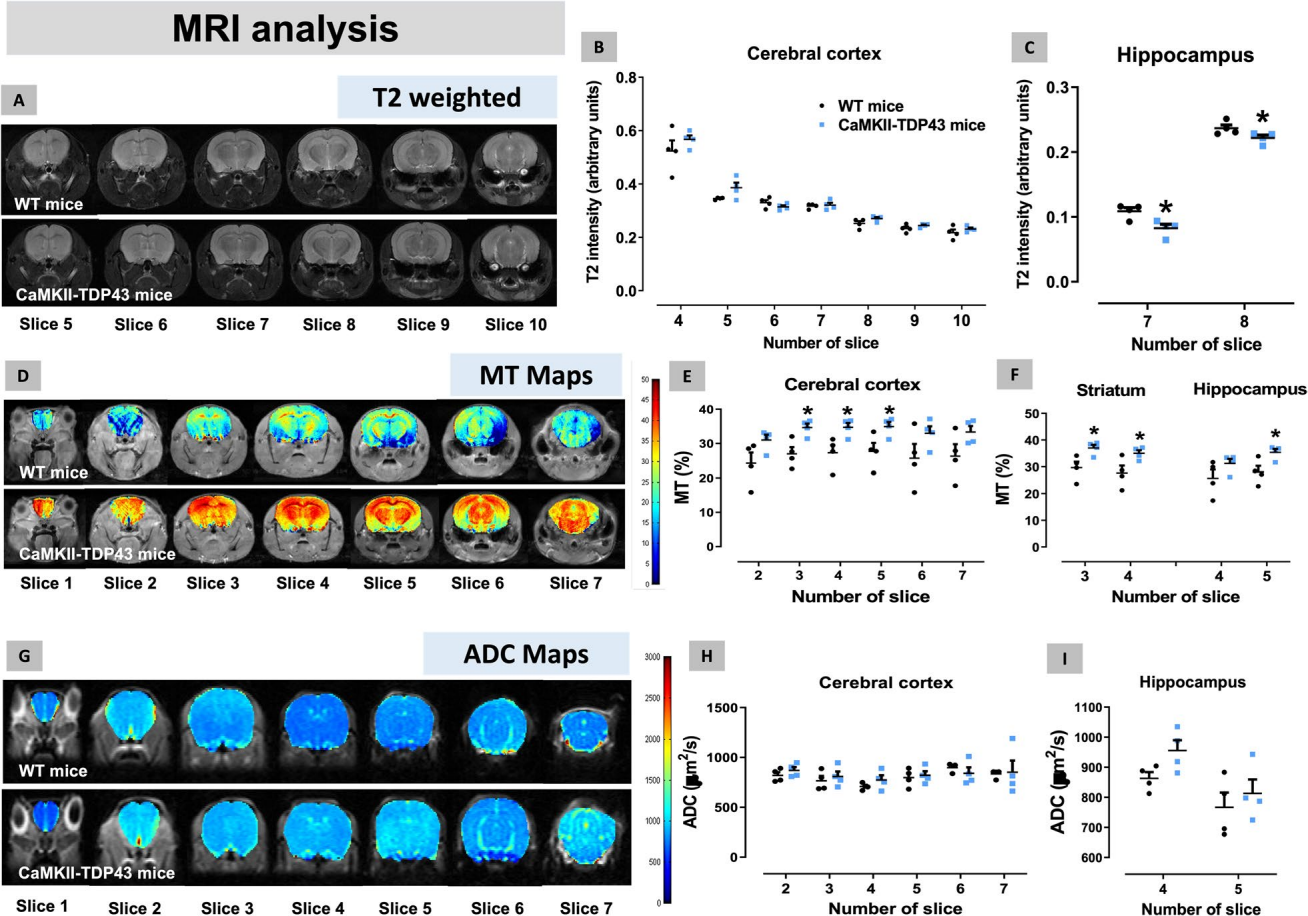
Data of T2 intensity (panels **A**–**C**), MT Maps (panels **D**–**F**) and ADC Maps (panels **G**–**I**) obtained by MRI analysis in several CNS structures of FTD and wildtype male mice at PND90. Details in the text. Data were expressed as means ± SEM and were analysed by Student’s t-test (*p < 0.05 versus wildtype mice)

We also performed brain MRI imaging analysis of FTD and wildtype mice at PND90 (Fig. 4A–I). The analysis of T2 volumetry allows the quantification of volume and thickness of a specific CNS structure, so that low values may reflect a possible atrophy. Our data demonstrated no changes in the cerebral cortex (Fig. 4A,B) and a small but statistically significant reduction in the hippocampus (Fig. 4A,C), similar to Tsai et al. [75] with mice of 6 months of age. The T2 intensity analysis of the whole brain indicated no volumetric differences between FTD and wildtype mice (data not shown). We also measured the percentage of MT, which may reflect the presence of oedema or signs of inflammatory events, and this parameter was found to be elevated in the cerebral cortex (Fig. 4D,E), as well as in the striatum and the hippocampus (Fig. 4D,F). Lastly, we recorded the ADC maps, which allows the detection of a possible alteration in the CNS parenchyma (e.g., presence of protein aggregates) that may counteract the movement of water molecules, but we did not find any differences between FTD and wildtype mice in both cortical (Fig. 4G,H and subcortical (hippocampus; Fig. 4G,I) structures, as well as in the whole brain (data not shown). In general, the data obtained from MRI analysis appear to indicate the occurrence of a marked hippocampal atrophy, accompanied by an inflammatory state that appears in those structures more directly affected in FTD, but that extends to other forebrain areas too.

As mice used for MRI analysis had been already subjected to behavioural testing, we wanted to analyse whether the imaging data correlated in FTD mice with the data of discrimination index obtained in the NOR test (Additional file 4: Fig. S4). Interestingly, we found a negative correlation between this index and the percentage of MT in the cerebral cortex (r = − 0.754; p < 0.05) and the hippocampus (r = − 0.745; p < 0.05) (Additional file 4: Fig. S4), thus indicating that lowest values of discrimination index (FTD animals having the worse performance in this test) corresponded with highest values for percentage of MT (FTD animals having highest oedema/inflammatory signs in these structures). In addition, a positive correlation between the discrimination index and the T2 intensity was found in the hippocampus (r = 0.877; p < 0.01), but not in the cerebral cortex (r = − 0.366, ns) (Additional file 4: Fig. S4), thus indicating that animals having the worse performance in this test also showed lowest values of T2 intensity reflecting greater atrophy.

### Neuropathological characterization of FTD mice: histopathological data

**Fig. 5:**
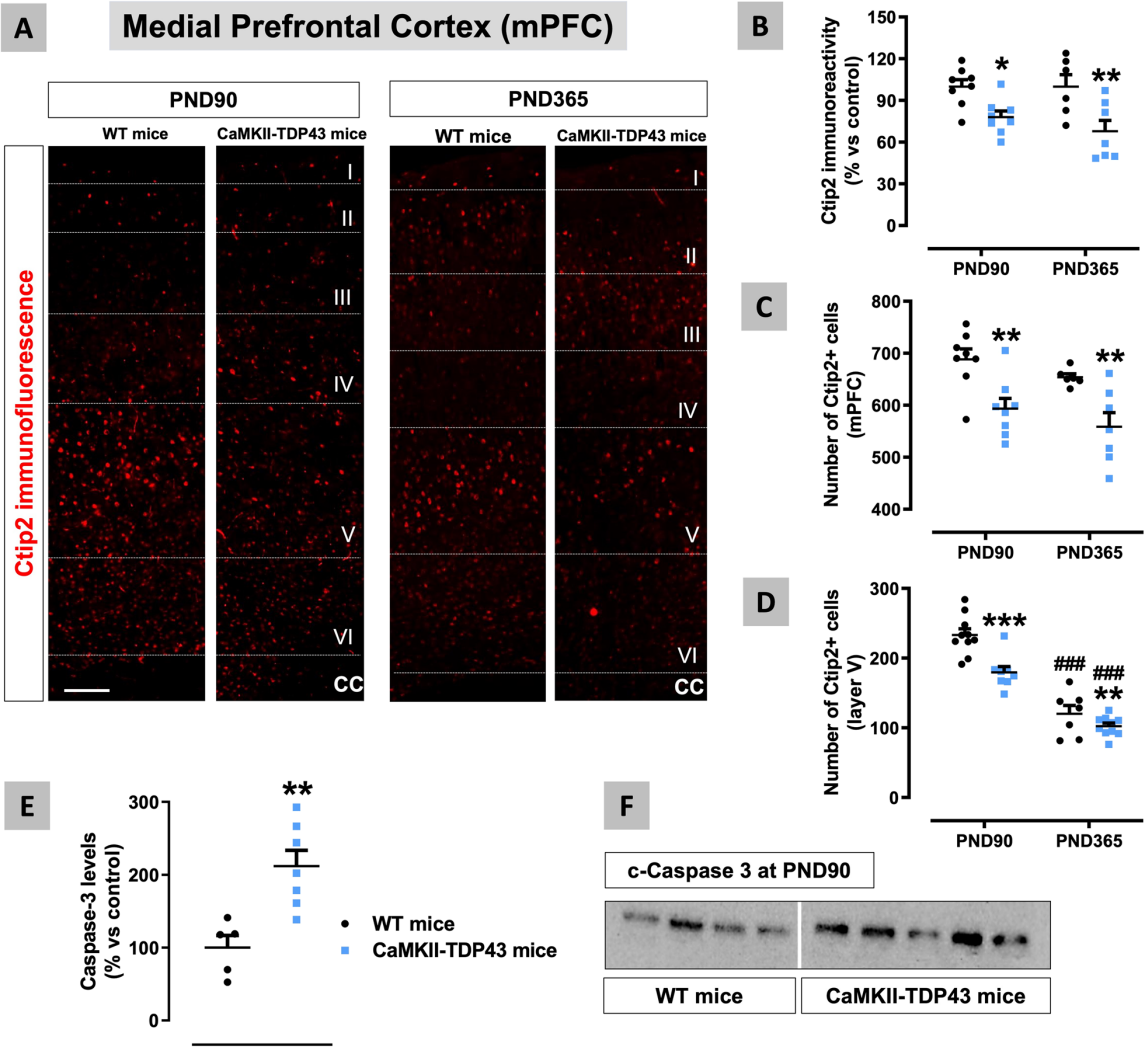
Data of Ctip2 immunofluorescence (panels **A–D**) in the mPFC of FTD and wildtype male mice at two different ages (PND90 and PND365), including representative microphotographs (panel **A**) for each genotype and the two ages investigated (scale bar = 50 μm), as well as data of caspase-3 levels at PND90 (panels **E**,**F**). Details in the text. Data were expressed as means ± SEM and were analysed by two-way analysis of variance followed by the Bonferroni test (*p < 0.05, **p < 0.01, ***p < 0.005 versus the corresponding wildtype mice; ###p < 0.005 versus the same group at PND90) for immunofluorescence, or the unpaired Student’s t-test (**p < 0.01) for Western blotting

**Fig. 6:**
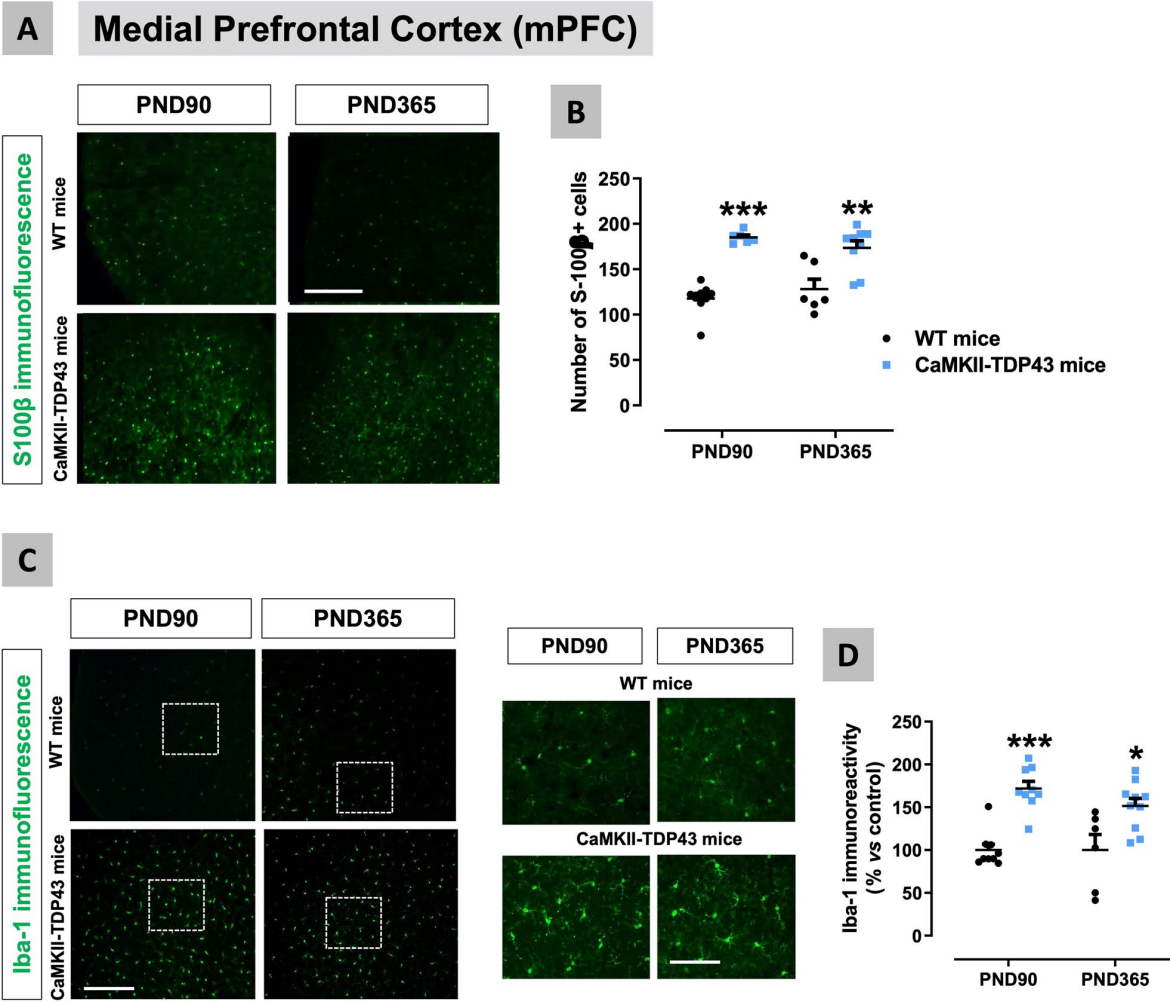
Data of S100β (panels **A**,**B**) and Iba-1 (panels **C**,**D**) immunofluorescence in the mPFC of FTD and wildtype male mice at two different ages (PND90 and PND365), including representative microphotographs (panels **A**,**C**) for each genotype and the two ages investigated (scale bar = 50 μmM (100 μm in inlets used for the analysis of some morphological characteristics of Iba-1-positive cells)). Details in the text. Data were expressed as means ± SEM and were analysed by two-way analysis of variance followed by the Bonferroni test (*p < 0.05, **p < 0.01, ***p < 0.005 versus the corresponding wildtype mice)

Next, we collected the brains of FTD and wildtype animals at the two ages of interest: PND90 and PND365, which were used for histopathological analysis in the two CNS structures of interest for FTD: the mPFC (located at the frontal lobe) and the hippocampus (located at the temporal lobe). Using immunofluorescence, we analysed first several markers of specific neuronal subpopulations in both CNS structures. Our data confirmed a reduction in the immunoreactivity for Ctip2 at PND90 that was maintained at PND365 (genotype: F(1,25) = 18.18, p < 0.0005; Fig. 5A,B). Such reduction also happened in the number of Ctip2-positive cells in the whole mPFC (genotype: F(1,25) = 21.24, p < 0.0001; Fig. 5A,C) and, in particular, in the layer V (genotype: F(1,18) = 17.26, p < 0.001; Fig. 5A,D), in which the pyramidal neurons that connect with subcortical areas are located, and this was observed again in these two structures at both PND90 and PND365 in FTD mice (Fig. 5A,C,D). However, in the last structure, the values at PND365 for both genotypes were lower compared with those measured at PND90 (age: F(1,13) = 129.8, p < 0.0001; Fig. 5D). Next, we investigated whether these neuronal losses found in the mPFC are associated with an elevated cell apoptosis, as described by Tsai et al. [75] who detected elevated levels of active caspase-3 in FTD mice. We also analysed levels of active caspase 3 (using an antibody against the Asp175 truncated form) in the mPFC in our FTD mice at PND90 which resulted to be significantly elevated (Fig. 5E,F). These neuronal losses were also associated in the mPFC of FTD mice at both PND90 and PND365 with elevated levels of S100β, a marker of astrocytes particularly recommended for this structure [88] (genotype: F(1,30) = 49.92, p < 0.005; Fig. 6A,B), and Iba-1, a marker of microglial cells (genotype: F(1,30) = 36.88, p < 0.0005; Fig. 6C,D). Reactive gliosis reflected in elevated GFAP levels was also described by Tsai et al. [75] in these mice at 6 months of age, so our data indicate that the inflammatory process would start at earlier stages (PND90). The analysis of some morphological aspects of microglial cells in the mPFC of FTD mice revealed that Iba-1-labelled cells had greater cell bodies and shorter branches (indicating an activated phenotype) compared to the smaller cell bodies and longer and thin processes (indicating a more quiescent phenotype) seen in wildtype mice (see inlets in Fig. 6C).

**Fig. 7:**
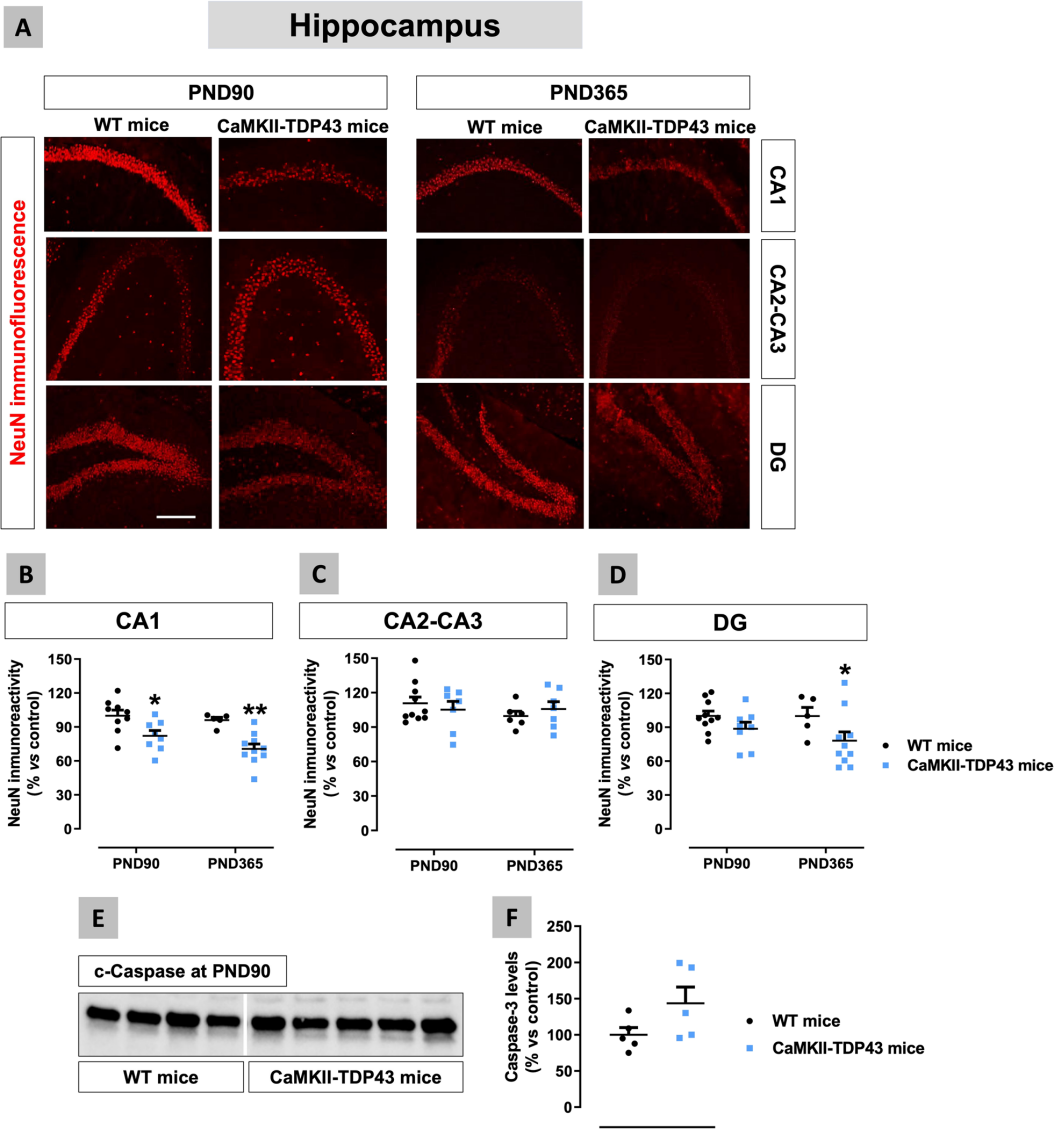
Data of NeuN immunofluorescence (panels **A**–**D**) in the hippocampus (CA1 and CA2–CA3 subfields, and dentate gyrus) of FTD and wildtype male mice at two different ages (PND90 and PND365), including representative microphotographs (panel **A**) for each genotype and the two ages investigated (scale bar = 50 μm), as well as data of caspase-3 levels at PND90 (panels **E**,**F**). Details in the text. Data were expressed as means ± SEM and were analysed by two-way analysis of variance followed by the Bonferroni test (*p < 0.05, **p < 0.01 versus the corresponding wildtype mice) for immunofluorescence, or the unpaired Student’s t-test for Western blotting

We also investigated different areas of the hippocampus in FTD mice, using NeuN immunostaining for labelling mature neurons, with relatively similar results (Fig. 7A–D). We found the maximal reduction in the CA1 subfield at PND90 (15%) and, in particular, at PND365 (30%) in FTD mice (genotype: F(1,17) = 19.79, p < 0.005; Fig. 7B), and lower effects in the dentate gyrus with statistical significance only at PND365 (genotype: F(1,29) = 5.923, p < 0.05; Fig. 7D). Therefore, in both structures, neuronal losses appear to be progressive from PND90 to PND365, and this correlates with the progression of the behavioural impairment. By contrast, no changes were seen at the CA2-CA3 subfields of FTD mice at both ages (genotype: F(1,15) = 0.001, ns; Fig. 7C). As in the mPFC, we also investigated whether hippocampal neuronal losses seen in FTD mice are associated with elevated levels of caspase-3, but, in this structure, which is a mixture of different subareas, ones having losses (CA1 and dentate gyrus) but others no (CA2-CA3), we only detected a trend towards an increase at PND90 (Fig. 7E,F).

**Fig. 8:**
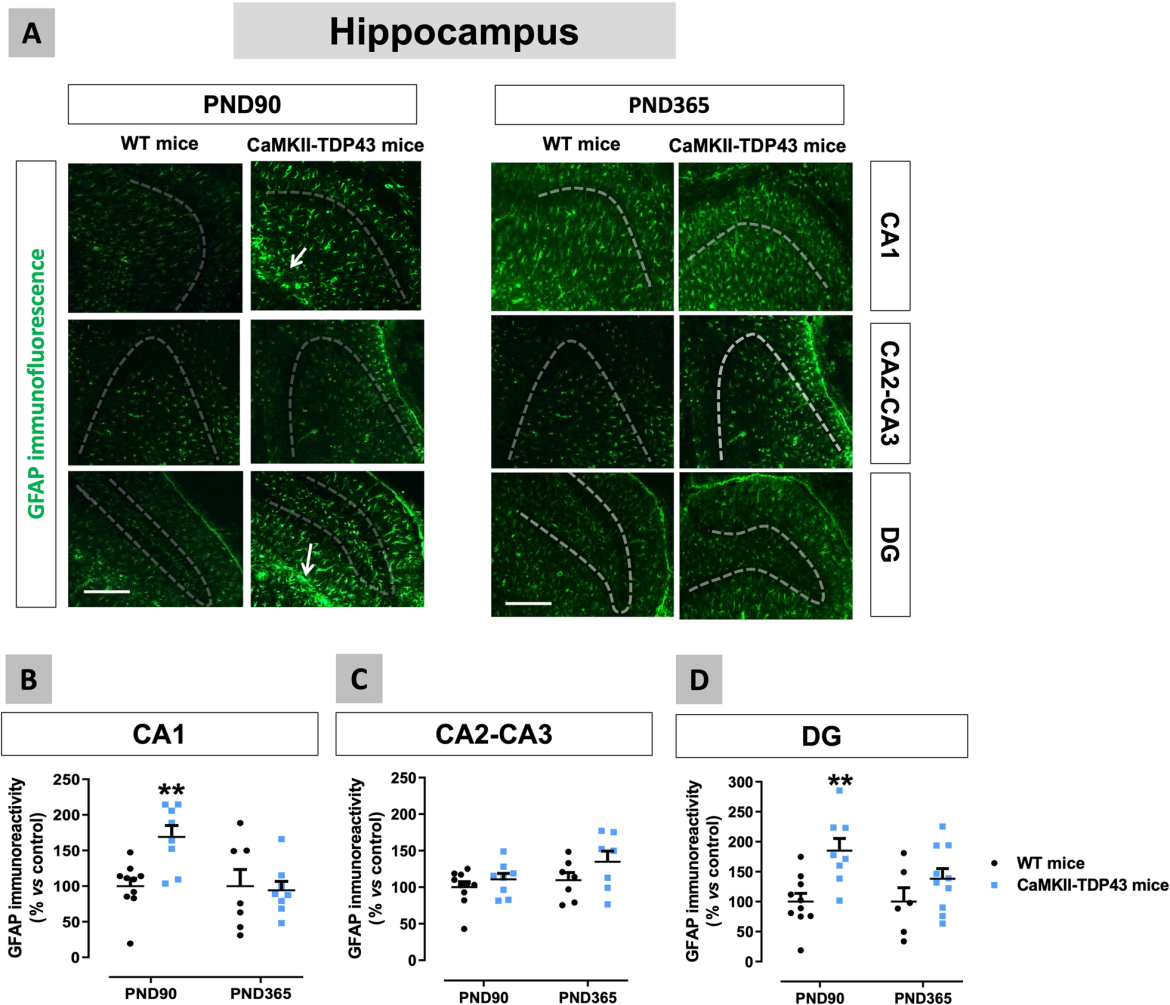
Data of GFAP immunofluorescence (panels **A**–**D**) in the hippocampus (CA1 and CA2–CA3 subfields, and dentate gyrus) of FTD and wildtype male mice at two different ages (PND90 and PND365), including representative microphotographs (panel **A**) for each genotype and the two ages investigated (scale bar = 50 μm) in which dotted lines and arrows indicate the position of the granular layer where the neuronal cell bodies and the glial scar are located. Details in the text. Data were expressed as means ± SEM and were analysed by two-way analysis of variance followed by the Bonferroni test (**p < 0.01 versus the corresponding wildtype mice)

**Fig. 9:**
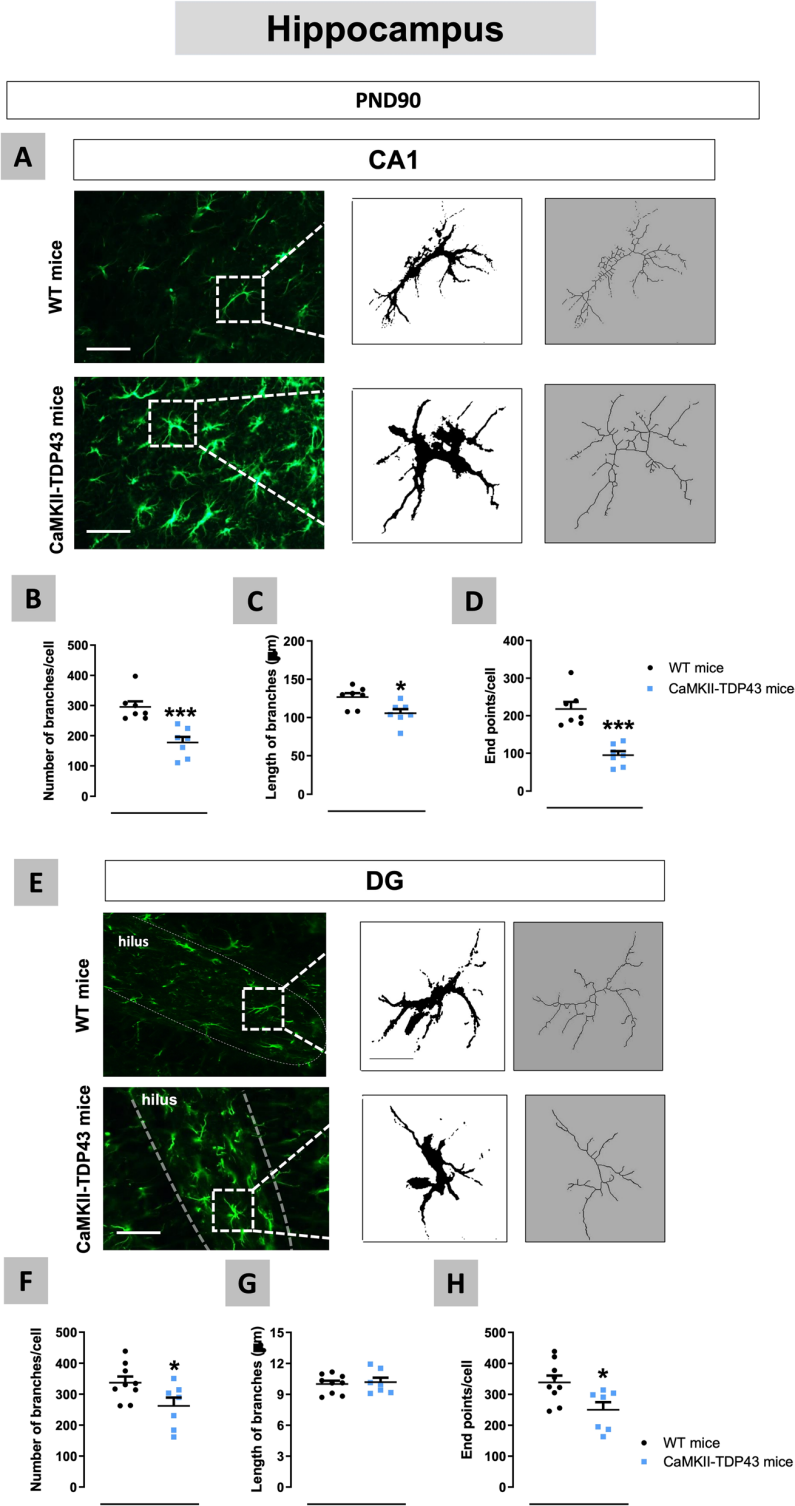
Morphological analysis (number and length of branches and counting of endpoints) of astrocytes, using GFAP immunofluorescence, in the hippocampus (CA1 (panels **A**–**D**) and dentate gyrus (panels **E**–**H**)) of FTD and wildtype male mice at PND90, including representative microphotographs (panels **A**,**E**) for each genotype and hippocampal region (scale bar = 100 μm and 500 μm). Details in the text. Data were expressed as means ± SEM and were analysed by Student’s t-test (*p < 0.05, ***p < 0.005 versus wildtype mice)

We also analysed the glial response in the hippocampus of FTD mice using first the astroglial marker GFAP (Fig. 8A–D), which resulted to be elevated in the CA1 subfield, but only at PND90 (genotype: F(1,29) = 4.201, p < 0.05; interaction: F(1,29) = 5.915, p < 0.05; Fig. 8B). A similar reactive astrogliosis was found in the dentate gyrus at PND90, with the formation of an intense glial scar (indicated with white arrows in the Fig. 8A), although, in this case, the data at PND365 showed certain trend to be elevated too (genotype: F(1,18) = 10.45, p < 0.01; interaction: F(1,18) = 2.025, ns; Fig. 8D). These smaller differences found in GFAP immunoreactivity at PND365 respect PND90 in the CA1 subfield (no differences between FTD and wildtype mice) and the dentate gyrus (a mere trend to be elevated) may be related to the elevation in this marker experienced by wildtype mice, which may be a physiological aging-related effect. This elevation was not visible in the Fig. 8B,D due to the data normalization (% over wildtype mice at each age), but it is evident with the analysis of raw data (not shown). By contrast with these two hippocampal areas, our data in the CA2-CA3 subfields proved no differences between genotypes and ages (Fig. 8C) in concordance with the data of NeuN immunostaining (Fig. 7C). As an extension of the above data, we also investigated the morphological characteristics of GFAP-positive cells (through the analysis of their cytoskeleton) at PND90 in the two hippocampal structures in which GFAP immunoreactivity was elevated in FTD mice (Fig. 9A,E). Our data indicated strong reductions in the number of branches (shorter and less ramified) (Fig. 9B) and endpoints *per* cell (Fig. 9D) and, to a lower extent, in the length of branches (Fig. 9C) in the CA1 subfield of FTD mice, thus supporting that astrocytes acquire in FTD mice a characteristic activated state. Similar responses, although to a lower extent, were found in the dentate gyrus (Fig. 9E–H), which is concordant with its lower degree of neuronal death.

**Fig. 10:**
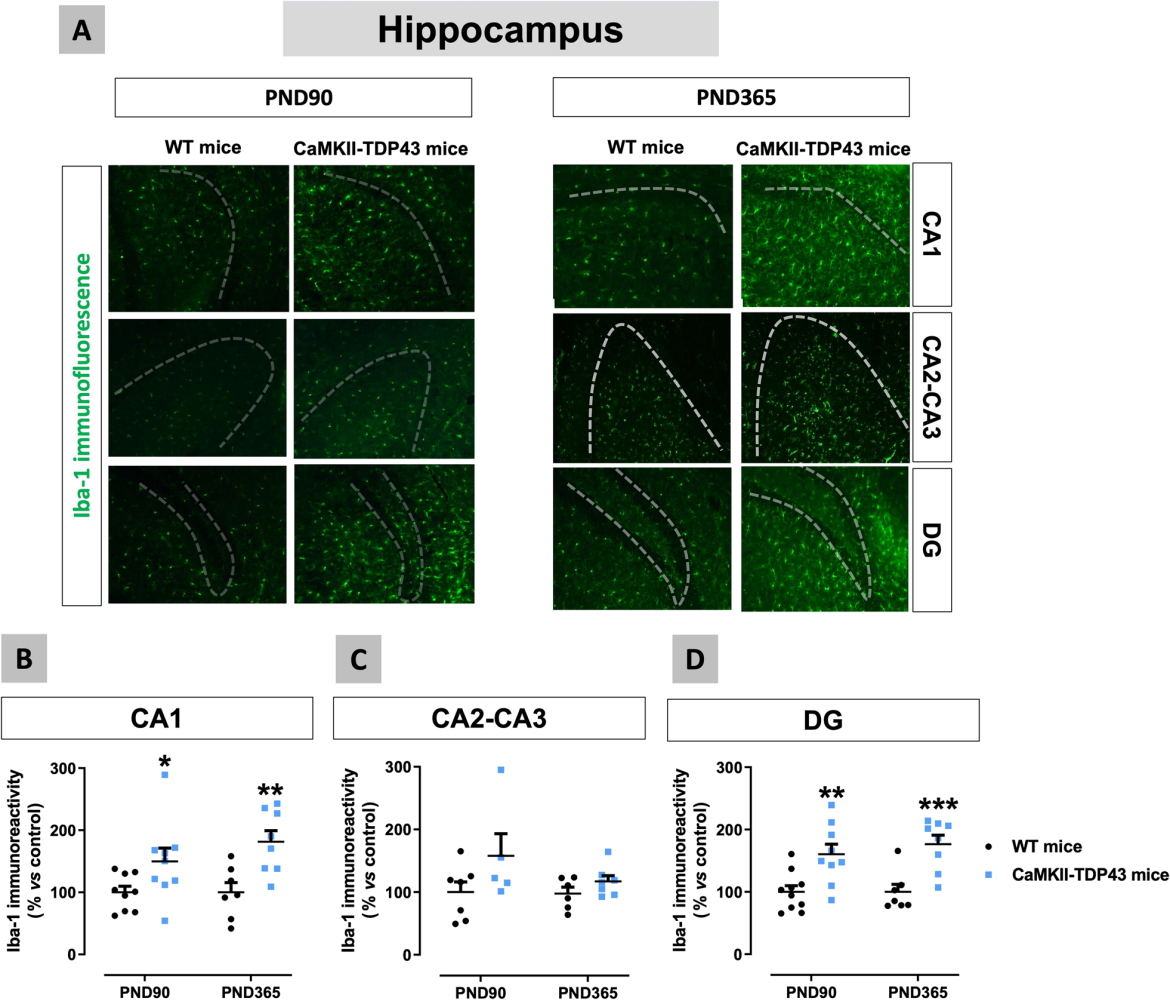
Data of Iba-1 immunofluorescence (panels **A**–**D**) in the hippocampus (CA1 and CA2–CA3 subfields, and dentate gyrus) of FTD and wildtype male mice at two different ages (PND90 and PND365), including representative microphotographs (panel **A**) for each genotype and the two ages investigated (scale bar = 50 μm), in which dotted lines indicate the position of the granular layer where the neuronal cell bodies are located. Details in the text. Data were expressed as means ± SEM and were analysed by two-way analysis of variance followed by the Bonferroni test (*p < 0.05, **p < 0.01, ***p < 0.005 versus the corresponding wildtype mice)

We also measured Iba-1 immunoreactivity, a marker of microglial cells, which resulted to be significantly elevated in the CA1 subfield (genotype: F(1,16) = 12.29, p < 0.01; Fig. 10A,B) and the dentate gyrus (genotype: F(1,17) = 18.97, p < 0.0005; Fig. 10A,D) at both ages, also revealing a more amoeboid morphology close to M1 phenotype in Iba-1-labelled cells (Fig. 10A). By contrast, only trends to be elevated (higher at PND90) were found in the CA2-CA3 subfield (genotype: F(1,21) = 4.458, p < 0.05; Fig. 10A,C), which again is concordant with the NeuN immunostaining data in this subfield (Fig. 7C). This parallelism between neuronal death in the different areas of the hippocampus and the microglial response (higher in areas of greater neuronal losses) supports an evident neurotoxic role played by these glial cells in our FTD mice.

As an additional analysis, and to confirm the above-described elevation in glial immunoreactivity and the changes in the morphological characteristics of these cells, we also measured by qPCR the levels of different markers of glial cells, such as the cytokines IL-1β and TNF-α, the glial glutamate transporter EAAT2 (GLT-1), and the M2 microglial marker arginase-1 in FTD and wildtype mice at the two ages selected for investigation: PND90 and PND365 (presented as Additional file 5: Fig. S5). Our data proved an elevation in IL-1β expression in FTD mice at PND90 in the mPFC and at PND365 in the hippocampus (p = 0.09), whereas the opposite changes (reduction in the mPFC at PND90 and in the hippocampus at PND365) were evident for arginase-1, possibly indicating a greater M1/A1 profile for microglial/astroglial cells at PND90 in the mPFC, but this occurred at PND365 in the hippocampus. No changes were detected for TNF-α at PND90, but a marked increase was evident in both structures at PND365, in concordance in part with the above comment. Lastly, the expression of EAAT2 was not altered in the mPFC at the two ages analysed, but an important increase was found in the hippocampus at PND365, again supporting the greater activated state of glial cells at this age in this CNS structure (Additional file 5: Fig. S5).

We have also investigated FTD mice at PND90 in relation with other additional aspect derived from the data of glial reactivity in the dentate gyrus, which may be related to the neurogenic activity of this structure. Given that GFAP immunostaining in this hippocampal structure may also label neural stem cells located at the subgranular layer [21], we carried out immunostaining with other marker of these cells as Sox-2, a transcription factor essential for the maintenance of self-renewal capacity of these neural stem cells. The objective was to be sure of the nature of GFAP-positive cells detected in the dentate gyrus (astrocytes or neural stem cells?). Our data indicated no differences in the number of Sox-2-positive cells in the dentate gyrus and also in the subgranular zone between FTD and wildtype mice at PND90 (Additional file 6: Fig. S6). In addition, we conducted double-labelling immunofluorescence between Sox-2 and GFAP and found a reduced ratio of colocalization in FTD mice, whereas Ki67 immunostaining, which is used as a marker of cell proliferation, indicated that this event, rather than being elevated, appeared to be lower in FTD mice (Additional file 6: Fig. S6). These data support that cells labelled with GFAP immunofluorescence in the dentate gyrus of FTD mice are more inflammatory astrocytes than neurogenic cells, as well as that these data indicated a reduced neurogenic response in the dentate gyrus of FTD mice that has been also found in patients [24]. Whether this reduced neurogenesis may have a role in cognitive deficits experienced by our FTD mice, as found in other studies [12, 31], will require additional research.

We also wanted to investigate whether the overexpression of TDP-43 in forebrain neurons of FTD mice, that results in its translocation to and aggregation in the cytosol [75], may be associated with alterations in protein degradation (UPS and autophagy). To this end, we analysed in FTD and wildtype mice the levels of some important proteins in this process, such as ubiquitin, p62 and the autophagy-related proteins LC3-I and LC3-II. Our data indicated elevated levels of ubiquitin and p62 in the mPFC of FTD mice (Additional file 7: Fig. S7), which may imply the occurrence of an elevated ratio of protein degradation. This correlated with fact that p-TDP-43/TDP-43 ratio was also elevated in this structure (see Table 1). Our data are concordant with studies that identified ubiquilin/p62-positive inclusions in post-mortem tissues of FTD (C9orf72-linked) patients [8, 50], and with others describing inclusions of TDP-43 with p62 [39]. However, in our study, no changes were detected for LC3-II and its ratio with LC3-I in both the mPFC and the hippocampus of FTD mice (Additional file 7: Fig. S7), suggesting similar autophagy flux in wildtype and FTD mice. This may explain why, despite the elevated labelling of proteins to be degraded, as reflected in the data of ubiquitin and p62 levels, the absence of an elevated autophagy flux may determine their accumulation in aggregates instead their complete elimination. Moreover, these changes were region-specific as they were not found in the hippocampus (Additional file 7: Fig. S7), where the ratio of p-TDP-43/TDP-43 in FTD mice was not so different compared to wildtype animals and also where the magnitude of neuronal deterioration was smaller (see above).

The last additional aspect that was investigated in the characterization of these FTD mice was the possibility that they may show characteristics of ALS at advanced ages, given the intimate relationship between both pathologies [26]. To this end, we analysed the ventral horn of the spinal cord of FTD and wildtype animals at PND365 using Nissl staining (to quantify the number of spinal motor neurons) and GFAP and Iba-1 immunostaining (to detect glial reactivity), and the data strongly indicated no differences between FTD and wildtype mice in any of these parameters (Additional file 8: Fig. S8), which is concordant with the general lack of motor effects in FTD mice at this longer age described above.

### Analysis of the endocannabinoid signaling in FTD mice

**Fig. 11:**
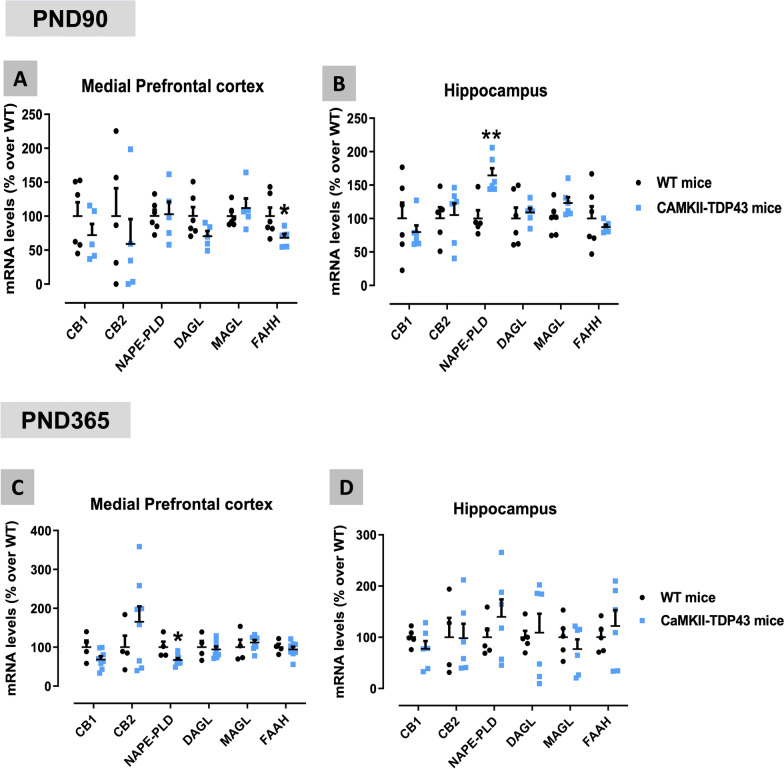
qPCR analysis of different endocannabinoid proteins (CB_1_ and CB_2_ receptors, NAPE-PLD, DAGL, MAGL and FAAH enzymes) in the mPFC (panels **A**,**C**) and the hippocampus (panels **B**,**D**) of FTD and wildtype male mice at two different ages (PND90 and PND365). Details in the text. Data were expressed as means ± SEM and were analysed by unpaired Student’s t-test (*p < 0.05, **p < 0.01 versus the wildtype mice)

**Fig. 12:**
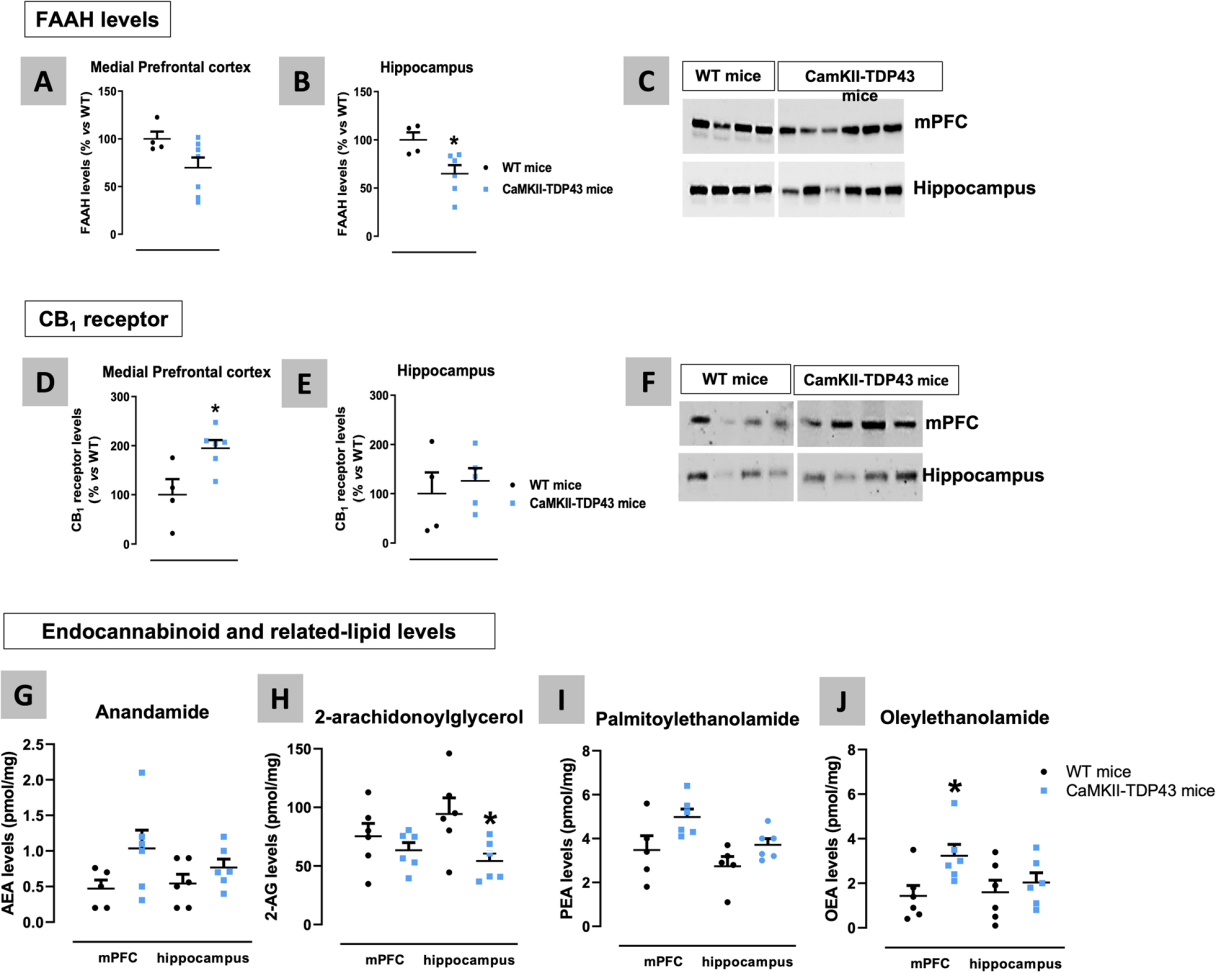
Western blot analysis of the FAAH enzyme (panels **A**–**C**) and the CB_1_ receptor (panels **D**–**F**), as well as LC-APCI–MS analysis of anandamide, 2-AG and related signaling lipids (panels **G**–**J**) in the mPFC and the hippocampus of FTD and wildtype male mice at PND90. Details in the text. Data were expressed as means ± SEM and were analysed by Student’s t-test (*p < 0.05 versus wildtype mice)

Our following objective was to analyse the status of specific elements of the endocannabinoid system in the two CNS structures of interest in these mice: mPFC and hippocampus. This may help to identify changes in this system that may have interest for FTD pathogenesis and/or therapeutic management. We first conducted a qPCR analysis which proved a decrease in the hydrolysing enzyme FAAH and the synthesizing enzyme DAGL (only as a trend: p = 0.091) in the mPFC of FTD mice at PND90 (Fig. 11A), accompanied by an increase in the synthesizing enzyme NAPE-PLD in the hippocampus also at PND90 (Fig. 11B). However, most of these changes disappeared at older ages (PND365) (Fig. 11C,D), although with some new alterations (reduction in NAPE-PLD and in CB_1_ receptor (only as a trend: p = 0.09) in the mPFC) appearing in FTD mice (Fig. 11C). All these endocannabinoid genes were also analysed in the cerebellum, a CNS structure in which, as mentioned above, TDP-43 was not overexpressed, and no differences between FTD and wildtype mice were detected (data not shown). We also analysed protein levels of some of these elements: FAAH and the CB_1_ receptor, using western blot, which confirmed the reduction in this enzyme in both the mPFC (trend: p = 0.082; Fig. 12A,C) and the hippocampus (Fig. 12B,C), and the elevation of the CB_1_ receptor in the mPFC (Fig. 12D,F), although these data were measured only at PND90. No changes were seen in the CB_1_ receptor in the hippocampus (Fig. 12E,F). Lastly, it is remarkable that the CB_2_ receptor was not altered in any of the two CNS structures analysed, neither at PND90 nor at PND365 (Fig. 11A–D), despite the important levels of glial reactivity (which are frequently associated with up-regulation of this receptor in other pathologies; see [22]) detected in these structures at these two ages.

We interpreted these changes in FTD mice, in particular those in FAAH, as part of an endogenous protective response aimed at elevating anandamide levels (and other related lipids) in these CNS structures. To confirm this, we analysed next the concentrations of endocannabinoids and related lipids in the two structures of interest in FTD and wildtype mice at PND90 (Fig. 12G–J). Our data reflected trends towards an increase in anandamide concentrations of FTD mice, in particular in the mPFC (p = 0.097; Fig. 12G), accompanied by similar responses in two related lipids that are also substrates of FAAH: PEA (p = 0.063 in the mPFC; p = 0.083 in the hippocampus; Fig. 12I) and OEA (p < 0.05 in the mPFC; Fig. 12J), which support such alterations as part of an endogenous protective response. By contrast, the concentrations of the other major endocannabinoid, 2-AG, proved a reduction (possibly related to the behavioural and histopathological alterations described above) in the hippocampus and, to a lower extent, in the mPFC (only as a trend; Fig. 12H).

### Neuroprotective properties of FAAH inhibition in FTD mice

**Fig. 13:**
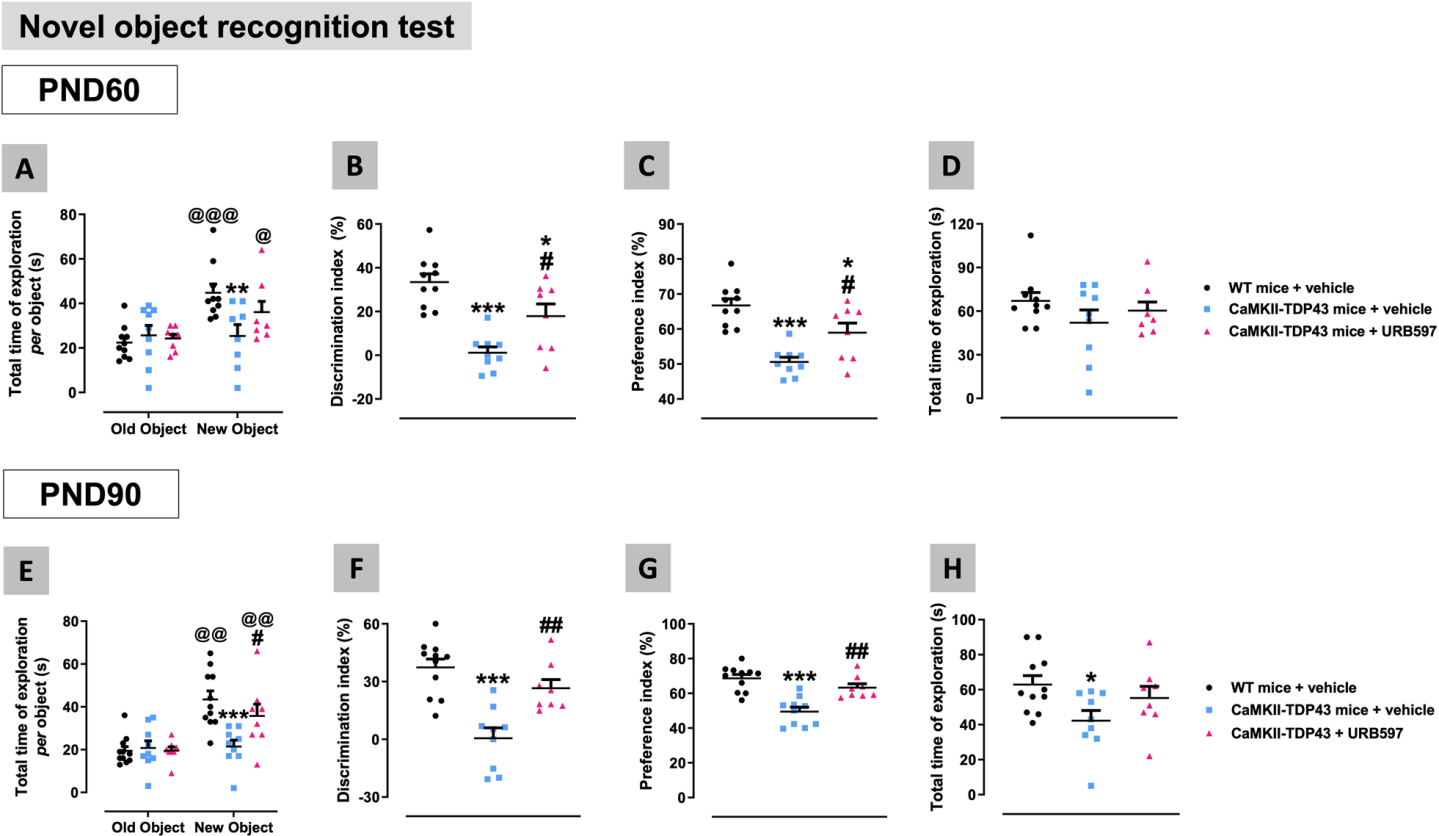
Behavioural data obtained in FTD and wildtype male mice at two different ages (PND60 (panels **A**–**D**) and PND90 (panels **E**–**H**)) in the Novel Object Recognition test after a chronic treatment with URB597 or vehicle. Details in the text. Data were expressed as means ± SEM and were analysed by one-way or two-way, as required, analysis of variance followed by the Bonferroni test (*p < 0.05, **p < 0.01, ***p < 0.005 versus wildtype mice treated with vehicle for each age; #p < 0.05, ##p < 0.01 versus FTD mice treated with vehicle for each age; @p < 0.05, @@p < 0.01, @@@p < 0.005 versus the same genotype and treatment for the exploration of the old object)

**Fig. 14:**
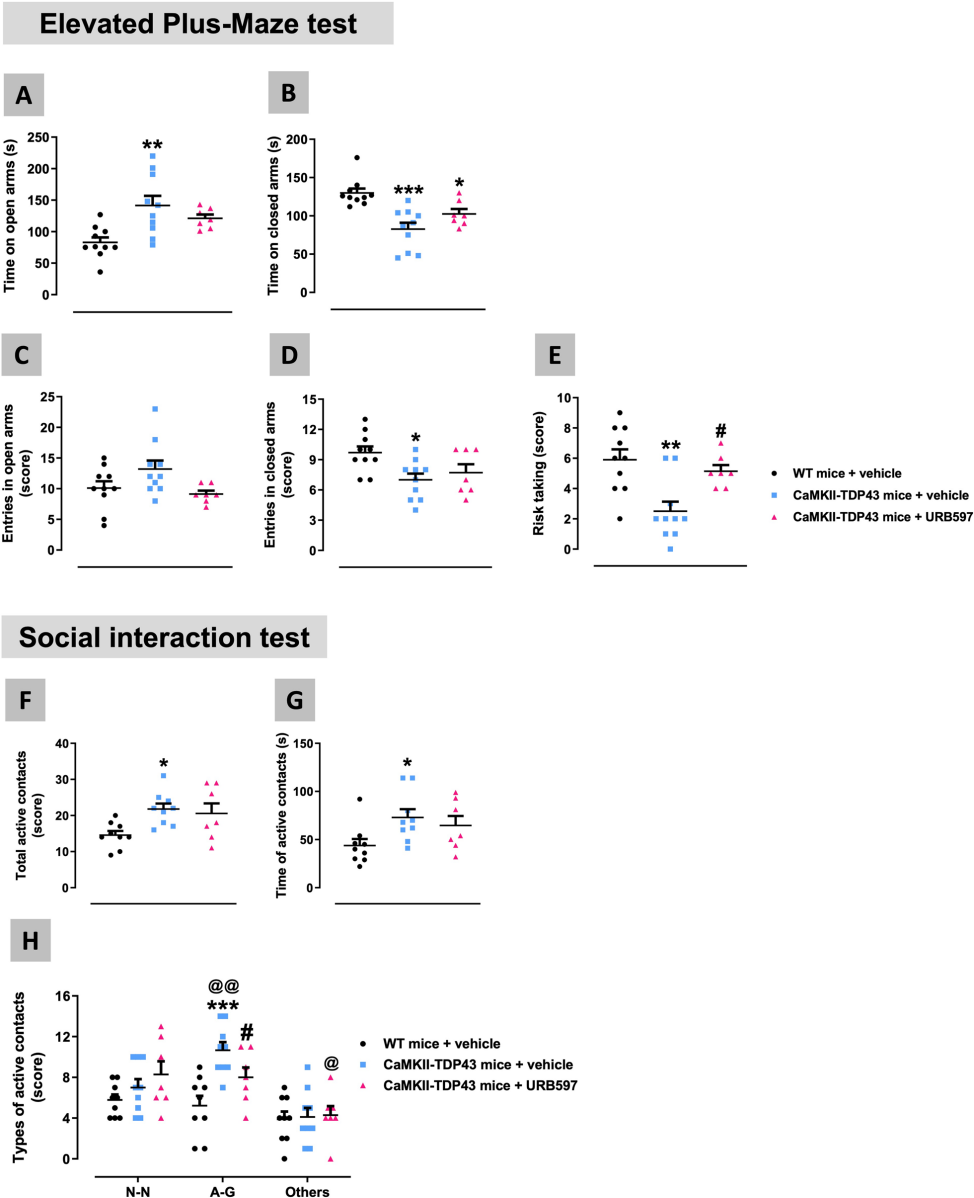
Behavioural data obtained in FTD and wildtype male mice at PND90 in the Elevated Plus Maze (panels **A**–**E**) and Social Interaction (panels **F**–**H**) tests after a chronic treatment with URB597 or vehicle. Details in the text. Data were expressed as means ± SEM and were analysed by one-way or two-way, as required, analysis of variance followed by the Bonferroni test (*p < 0.05, **p < 0.01, ***p < 0.005 versus wildtype mice treated with vehicle; #p < 0.05 versus FTD mice treated with vehicle; @p < 0.05, @@p < 0.01 versus the same genotype and treatment for each type of active contacts)

Assuming that changes in FAAH and endocannabinoid concentrations may be part of an endogenous protective response elicited by the neurodegenerative process in FTD mice, our next experiment was aimed at investigating the effects of the pharmacological inactivation of this enzyme, a treatment that is well-documented to elevate anandamide levels [59], which may enhance such endogenous protective response. This was investigated using the selective FAAH inhibitor URB597 administered to FTD mice from pre-symptomatic phases (PND45) to symptomatic stages (PND90), and analysing the behavioural status of these mice at PND60 and PND90, as well as those histopathological markers that were found to be altered in the progression of FTD at PND90. First, we analysed the potential benefits of URB597 against cognitive deterioration shown by FTD mice in the NOR test (Fig. 13A–H). Our data showed a recovery after the pharmacological FAAH inhibition in the low values for the discrimination (PND60: F(2,24) = 16.75, p < 0.0001; see Fig. 13B; PND90: F(2,25) = 16.14, p < 0.0001; see Fig. 13F) and preference (PND60: F(2,24) = 16.75, p < 0.0001; see Fig. 13C; PND90: F(2,26) = 18.78, p < 0.0001; see Fig. 13G) indices shown by FTD mice. This was also evident for the time shown by animals in exploring the objects at the two ages (Fig. 13D,H), in particular the new object, whose values were significantly lower in FTD mice, but recovered values similar to controls after the treatment with URB597 (2-way interaction: PND60: F(2,23) = 6.823, p < 0.005; see Fig. 13A; PND90: F(2,25) = 10.97, p < 0.0005; see Fig. 13E). These beneficial effects of URB597 were also found for some of the impairments experienced by FTD mice in the elevated plus maze test at PND90; for example, the compound reduced the elevated time (F(2,24) = 7.425, p < 0.005; Fig. 14A) and, to a lower extent, the number of entries (F(2,24) = 3.233, p = 0.0571; Fig. 14C) in open arms, whereas it increased (reduction in probability levels compared to wildtype mice) the low values for the time (F(2,24) = 12.26, p < 0.0005; Fig. 14B) and the number of entries (F(2,24) = 4.743, p < 0.05; Fig. 14D) in close arms. Such benefits were also evident, even to a greater extent (reaching values of wildtype mice), in the risk taking measured in this test (Fig. 14E), which, as mentioned above, serves as a sign of impulsivity. Thus, our data indicated a reduced score for this parameter in FTD mice compared to wildtype animals, reflecting higher impulsivity (in concordance with their greater presence in open arms), which was significantly attenuated after the treatment with URB597 (F(2,24) = 8.682, p < 0.005; Fig. 14E), meaning that URB597-treated FTD mice explored more the open arms without leaving the closed arms, which reflect lower impulsitivity. However, the effects of URB597 were much more limited against the impaired behaviour shown by FTD mice in the social interaction test at PND90, with very modest trends towards a reduction in the number (F(2,22) = 4.858, p < 0.05; Fig. 14F) and time (F(2,22) = 3.462, p < 0.05; Fig. 14G) in active contacts, only reaching statistical significance for the anogenital contacts (interaction: F(4,44) = 3.865, p < 0.01; Fig. 14H).

**Fig. 15:**
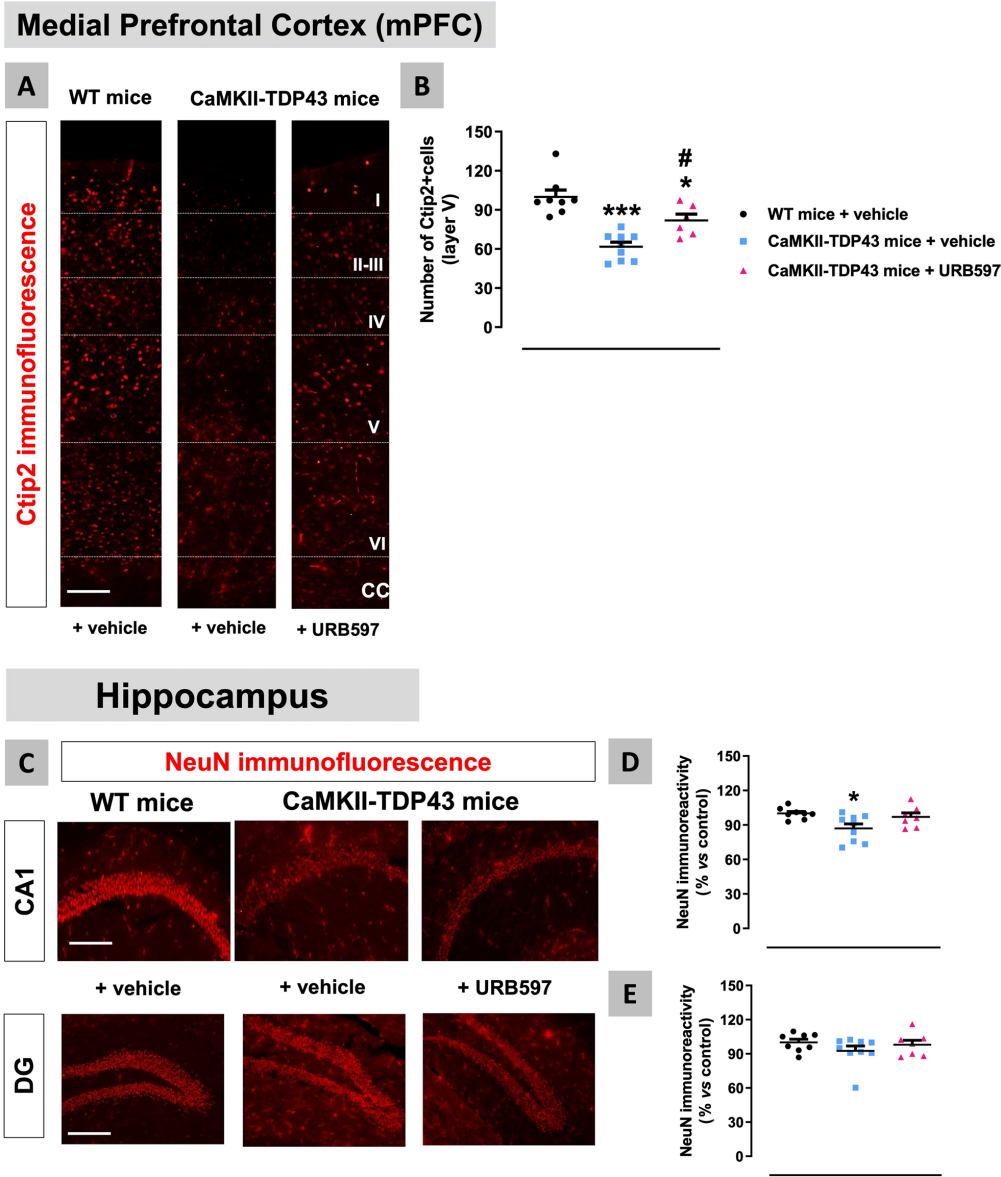
Data of immunofluorescence with Ctip2 in the mPFC (panels **A**,**B**) and NeuN in the hippocampus (CA1 subfield and dentate gyrus) (panels **C**–**E**) of FTD and wildtype male mice at PND90 after a chronic treatment with URB597 or vehicle, including representative microphotographs (panels **A**,**C**) for each genotype and treatment (scale bar = 50 μm). Details in the text. Data were expressed as means ± SEM and were analysed by one-way analysis of variance followed by the Bonferroni test (*p < 0.05, ***p < 0.005 versus wildtype mice treated with vehicle; #p < 0.05 versus FTD mice treated with vehicle)

These beneficial effects of URB597 observed at the behavioural level are presumably related to its capability to preserve pyramidal neurons in the mPFC and the hippocampus (Fig. 15A–E). Our analyses indicated a partial recovery in the number of Ctip2-positive cells in the layer V (F(2,20) = 19.96, p < 0.0001) after the treatment with URB597 (Fig. 15A,B). The same effect was found, using NeuN immunostaining, in the hippocampal CA1 subfield although to a much lower intensity (F(2,21) = 4.672, p < 0.05; Fig. 15C,D), and was not visible in the dentate gyrus (Fig. 15C,E) in this cohort, in which NeuN immunostaining was poorly affected in FTD mice treated with vehicle (F(2,21) = 1.084, ns), as also found in the first experiment where only a trend towards to be reduced was evident.

**Fig. 16:**
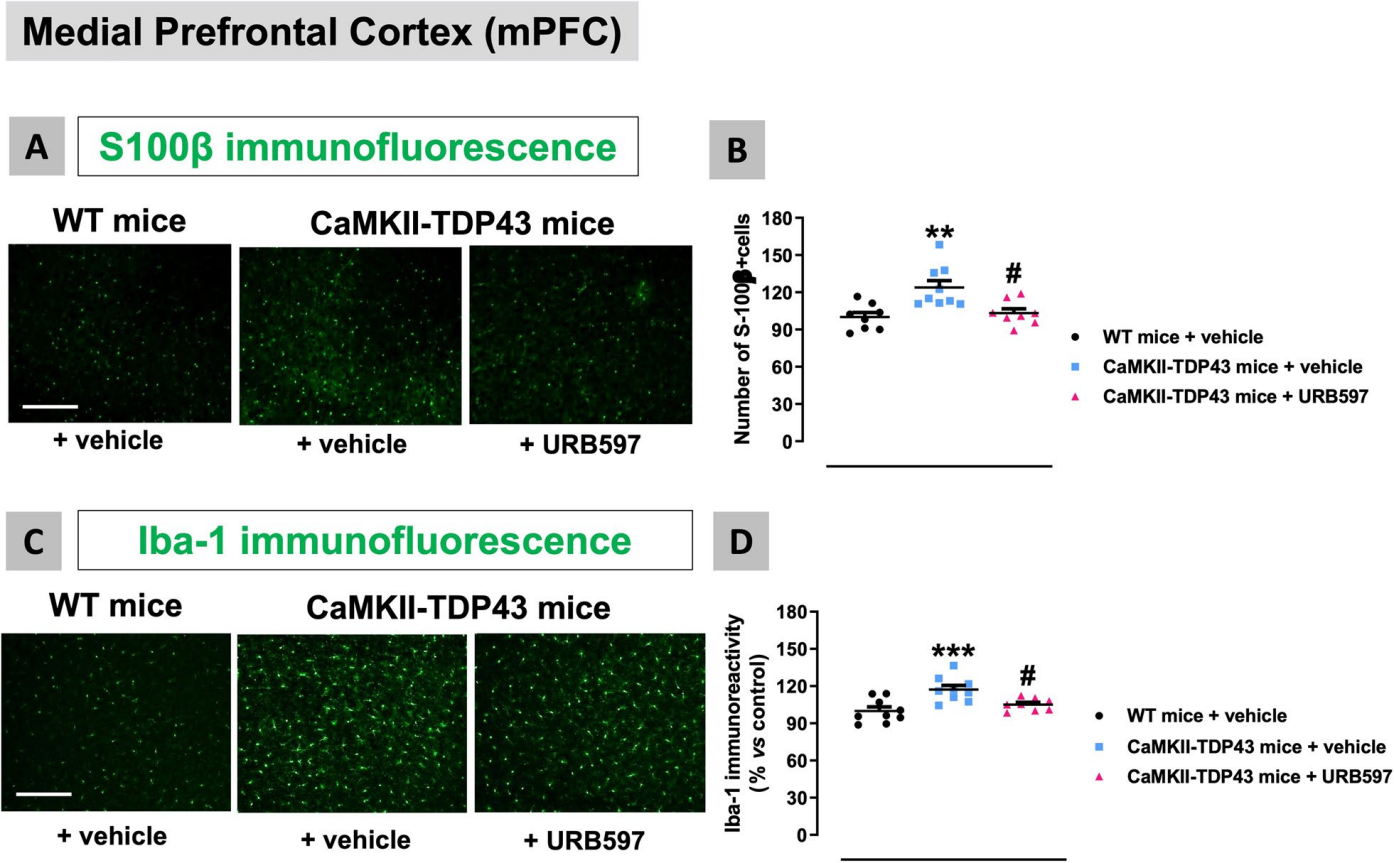
Data of S100β (number of positive cells; panels **A**,**B**) and Iba-1 (immunoreactivity levels; panels **C**,**D**) immunofluorescence in the mPFC of FTD and wildtype male mice at PND90 after a chronic treatment with URB597 or vehicle, including representative microphotographs (panels **A**,**C**) for each genotype and treatment (scale bar = 50 μm). Details in the text. Data were expressed as means ± SEM and were analysed by one-way analysis of variance followed by the Bonferroni test (**p < 0.01, ***p < 0.005 versus wildtype mice treated with vehicle; #p < 0.05 versus FTD mice treated with vehicle)

**Fig. 17:**
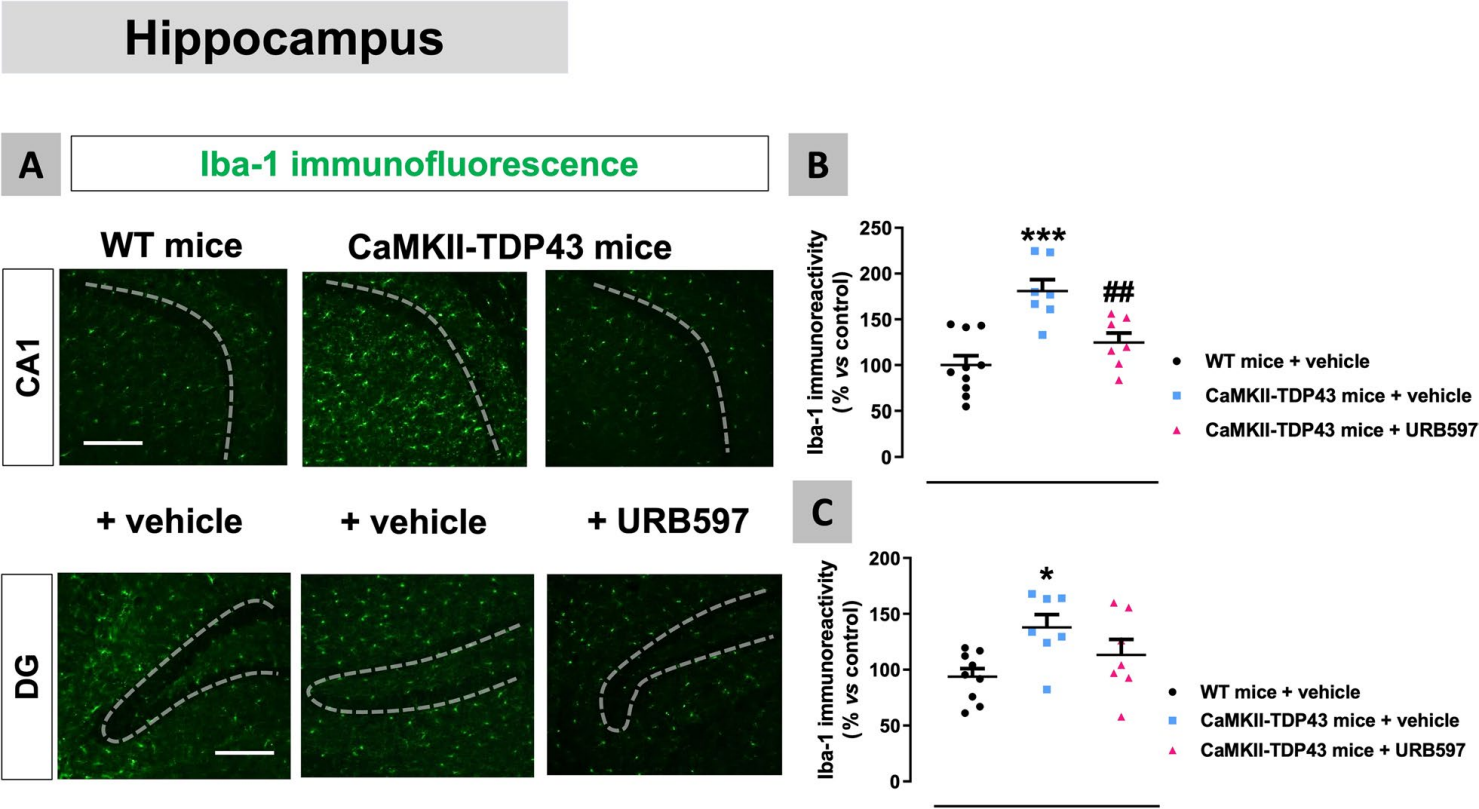
Data of Iba-1 immunofluorescence in the hippocampus (CA1 subfield and dentate gyrus) (panels **A**–**C**) of FTD and wildtype male mice at PND90 after a chronic treatment with URB597 or vehicle, including representative microphotographs (panel **A**) for each genotype and treatment (scale bar = 50 μm) in which dotted lines indicate the position of the granular layer where the neuronal cell bodies are located. Details in the text. Data were expressed as means ± SEM and were analysed by one-way analysis of variance followed by the Bonferroni test (*p < 0.05, ***p < 0.005 versus wildtype mice treated with vehicle; ##p < 0.01 versus FTD mice treated with vehicle)

**Fig. 18:**
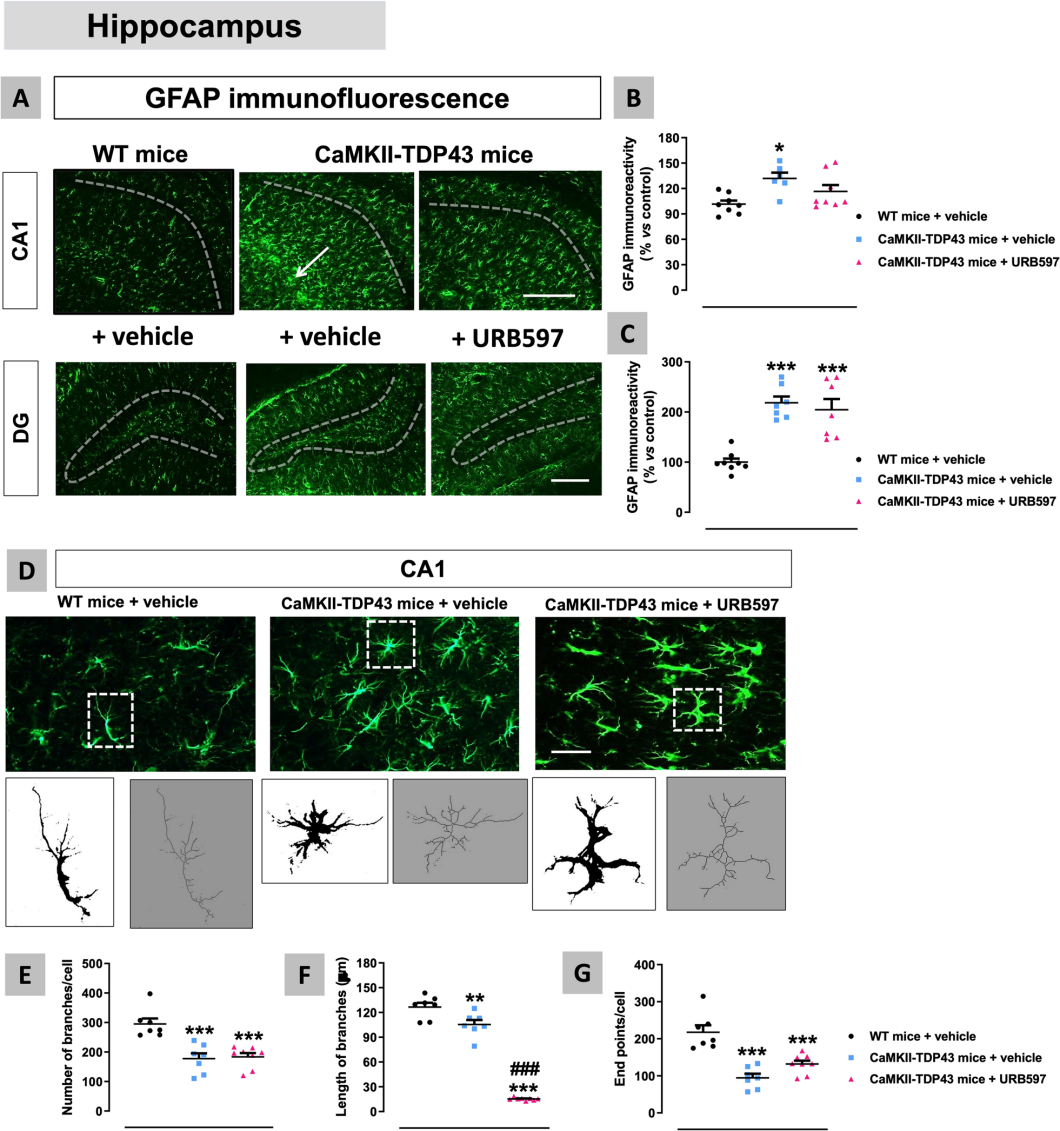
Data of GFAP immunofluorescence (panels **A**–**C**) and analysis of morphological characteristics (number and length of branches and counting of endpoints; panels **D**–**G**)) of GFAP-positive cells in the hippocampus (CA1 subfield and dentate gyrus) of FTD and wildtype male mice at PND90 after a chronic treatment with URB597 or vehicle, including representative microphotographs (panels **A**,**D**) for each genotype and treatment (scale bar = 50 μm, except in microphotographs for the morphological analysis in which they were 100 or 500 μm), in which dotted lines indicate the position of the granular layer where the neuronal cell bodies and the glial scar are located. Details in the text. Data were expressed as means ± SEM and were analysed by two-way analysis of variance followed by the Bonferroni test (*p < 0.05, ***p < 0.005 versus wildtype mice treated with vehicle; ###p < 0.005 versus FTD mice treated with vehicle)

This neuronal preservation in the mPFC caused by URB597 is associated with a strong reduction of neuroinflammatory events derived from reactive gliosis. Thus, we found a complete normalization by FAAH inhibition in the number of cells positive for the astrocyte marker S100β (F(2,22) = 8.676, p < 0.005; Fig. 16A,B), as well as in the immunoreactivity for the microglial marker Iba-1 (F(2,23) = 9.67, p < 0.001; Fig. 16C,D), which were significantly elevated in FTD mice, as shown before and again in this experiment. We also analysed glial reactivities in the hippocampus after the treatment with URB597, with relatively similar results to those found in the mPFC against microglial reactivity, despite the neuronal losses in FTD and recoveries after FAAH inhibition were much less intense in the different hippocampal subareas. Thus, we observed an important reduction by FAAH inhibition in the elevated levels of Iba-1 immunoreactivity found in FTD mice in both the CA1 subfield (F(2,21) = 13.82, p < 0.0001; Fig. 17A,B) and, to a lower extent (only a trend with the posthoc analysis), the dentate gyrus (F(2,20) = 4.401, p < 0.05; Fig. 17A,C), which correlates with the fact that neuronal loss almost did not exist in this hippocampal subarea in this experiment. A more detailed analysis of Iba-1-positive cells confirmed that these reductions are related to both lower immunoreactivity levels and also lower number of Iba-1-positive cells (data not shown). As regards to GFAP immunoreactivity, our data indicated a similar response in the CA1 subfield (F(2,19) = 5.567, p < 0.05; Fig. 18A,B) as for Iba-1, but no recovery in the case of the dentate gyrus (Fig. 18A,C) again in concordance with its data of NeuN immunostaining. In the CA1 subfield, we also analysed the morphological characteristics of GFAP-positive cells based on the examination of their cytoskeleton (see Fig. 18D). They acquired a strong activated (“ameboid”) state (less and shorter branches, and endpoints per cell) in FTD mice, but the treatment with URB597 did not alter this state, even caused a greater reduction in the length of branches (F(2,19) = 213.4, p < 0.0001; see Fig. 18E–G). This paradoxical effect may be related, as will be addressed later, to a greater metabolism of anandamide (possibly elevated by FAAH inhibition with URB597) by COX-2 in these cells, thereby generating oxygenated derivatives (prostamides) with a cannabinoid receptor-independent proinflammatory and neurotoxic profile [37]. This may promote astrogliosis as found in our morphological analysis in this hippocampal structure, thus opposing the expected neuroprotective effect of anandamide exerted by activation of cannabinoid receptors, which may explain the lower neuronal preservation found with URB597 in hippocampal areas.

Lastly, we also analysed the effects of URB597 in a few wildtype individuals at both behavioural and histopathological levels, but these data did not differ from those observed in wildtype animals treated with vehicle (see Additional file 9: Fig. S9), thus indicating that the beneficial effects of URB597 were selective for FTD mice in this experiment.

## Discussion

This is the first study investigating the endocannabinoid system and the neuroprotective potential of endocannabinoids in TDP-43-dependent FTD, using an experimental model in mice. The neuroprotective potential of different cannabinoids and other modulators of the endocannabinoid system have been largely investigated in the last 15–20 years, mainly in preclinical models, in other chronic progressive neurodegenerative disorders (reviewed in [14, 22]), including TDP-43-based models of ALS [18, 19, 66], and also in Tau-dependent models of FTD [10, 25]. To this end, we have used a murine model of TDP-43-dependent FTD, generated by Dr. Shen’s group [75], based on the overexpression of TDP-43 in forebrain structures in which those neuronal subpopulations more affected in FTD are located. Our first objective was to confirm that this model recapitulates in male mice the major behavioural characteristics of FTD, as demonstrated in Tsai et al. [75], which include deficits in domains associated with the generation of short- and long-term spatial, working and recognition memory. These alterations also extend to decision making and are accompanied by impairment in mood-like signs, disinhibition of social behaviour, and other behavioural changes, with no alterations in motor activity, which excludes that this model may develop long-term signs of ALS too, although this will still require further examination in female mice. We have confirmed that these behavioural abnormalities appear at early adulthood (PND90) and are associated with neuronal loss (likely caused by TDP-43 aggregation in forebrain neurons which triggers different neurotoxic events) and glial reactivity occurring at two key CNS structures: the mPFC and  the hippocampus, which play important regulatory roles in the brain functions mentioned above (e.g., cognitive processes, social interaction, emotional responses) that are altered in FTD. These two structures interact in a synchronic and coordinated manner to regulate important cognitive domains [69]. Pyramidal neurons located in the layer V of the mPFC (labelled with Ctip-2) and in the CA1 subfield of the hippocampus (labelled with NeuN) are of particular relevance in this case, as both groups of neurons play a key role in the maintenance of the bidirectional communication between the mPFC and the hippocampus [35, 63], which may explain their early degeneration compared to pyramidal neurons located in the dentate gyrus, which are affected later.

A second important result of our study was to confirm that the major behavioural and histopathological abnormalities characteristics of these mice still persisted at ages (PND365) older than those investigated by Tsai et al. [75], or even that some new abnormalities occur predominantly at these later ages, thus recapitulating disease progression in FTD patients [17, 53]. For example, in the elevated plus-maze and the tail suspension tests, signs of apathy, anhedonia or depression appear at later ages (PND365) to follow the signs of impulsivity more evident at PND90. It is important to remark that apathy, depression or anhedonia have been also found in other FTD murine models, for example those based on overexpression of mutant Tau (also under the CaMKIIα promoter) or in *GRN*-deficient mice that show cytosolic TDP-43 aggregates [23, 40]. In addition, these models also recapitulate the remaining behavioural impairments, mainly those related to cognitive deterioration [28, 67, 74]. The possibility to investigate these mice at older ages has also allowed the analysis of animal survival, demonstrating that FTD mice experience a premature death with all animals dying before PND365.

An important aspect of our study is that it explores not only the neuronal loss in the two key CNS structures for FTD, but also the possible contribution of glial reactivity to this loss through the so-called non-autonomous cell death [44]. Our data showed important levels of gliosis (elevated immunoreactivity and more glial cells, in particular those having an activated morphology) starting from the early stages in the disease (PND90) and accompanying the reduction in neuronal markers (Ctip-2, NeuN), which were also evident with MRI procedures. This confirms the contribution of activated astrocytes and microglial cells to the death of cortical and hippocampal pyramidal neurons in FTD mice. This aspect has been also investigated in patients using post-mortem tissues [42] or with PET imaging [52], which provided evidence of gliosis in the frontal cortex and the hippocampus, associated with elevated levels of proinflammatory cytokines in CSF and plasma [71, 85].

The next objective of our study was to explore whether the endocannabinoid signaling system in these two key CNS structures may become dysregulated (or simply altered), as in other neurological disorders [14], and whether such modifications may help to understand how they contribute to the FTD pathogenesis and/or how they could be used for a better design of neuroprotective treatments. In this case, our results indicate the occurrence of changes affecting the balance between synthesis and degradation of endocannabinoids, in particular anandamide, a fact in part confirmed by the analysis of concentrations of this endocannabinoid and other related *N*-acylethanolamines (e.g. PEA, OEA) regulated by the same pathways, which tended to be elevated in these two key CNS structures. Our interpretation of these changes is that they may serve as an endogenous response aimed at counteracting the progression of the neurodegenerative process, as it has been found in other neurodegenerative disorders. For example, in an experimental model of ALS based on overexpression of mutant TDP-43, FAAH expression was reduced in the spinal cord in parallel to elevated levels of 2-AG [18], whereas NAPE-PLD and the levels of anandamide resulted to be increased also in the spinal cord of the ALS murine model based on mutant SOD-1 [5, 54, 84]. Similar modifications in endocannabinoid and endocannabinoid-like mediator regulating enzymes resulting in altered levels of these lipids have been described in other neurodegenerative disorders (reviewed in [14]). However, our data did not demonstrate relevant changes in cannabinoid receptors, in particular in CB_2_ receptors, which, as mentioned above, are frequently up-regulated in glial elements when the latter become reactive in most of neurodegenerative and neuroinflammatory disorders [22]. This happened despite the occurrence of strong reactive gliosis in the two CNS structures altered in our FTD mice, so more research will be necessary to elucidate the reasons for this lack of elevated CB_2_ receptor expression.

Our last objective was to explore whether inducing local elevations of anandamide (and its congeners PEA and OEA) by inhibiting FAAH with the selective inhibitor URB597 [59], may have beneficial effects in delaying the progression of the pathological phenotype in FTD mice. Such strategy has been already used for other neurological disorders with promising results [1, 11, 51, 73, 81, 82] and the fact that, in our study, FAAH enzyme experiences in FTD mice a reduction associated with trends of anandamide, PEA and OEA to be elevated, supports its pharmacological interest. Our results confirmed the benefits of FAAH inhibition, since we found significant improvements in the behaviours altered in FTD mice after treatment with URB597, as well as in the loss of pyramidal neurons and associated astroglial and microglial reactivities visible in the mPFC (URB597 reduced both astrogliosis and microgliosis), and the hippocampus (the reduction was restricted to microgliosis). Such neuroprotective effects are concordant with results of studies with other experimental models of different neurodegenerative disorders, conducted mainly with FAAH inhibitors but also with modulators of other endocannabinoid-related enzymes (reviewed in [77]). As regards to the mechanism(s) that may underlie these neuroprotective effects, we assume that the URB597-induced elevation of anandamide should be the expected option, as demonstrated in previous studies using this FAAH inhibitor [59]. This elevation would occur locally at specific synapses or glial cells which may enhance anandamide activity at the classic cannabinoid receptors possibly located in neighboring cells and whose activation has demonstrated to have important benefits for cell survival and integrity [22]. In this sense, some studies have suggested that the effects of a local elevation of anandamide levels induced by URB597 would be predominantly exerted by the activation of CB_1_ receptors [29, 59], since the highest levels of FAAH are found in those CNS areas in which the CB_1_ receptor is also more concentrated (e.g., neocortex, hippocampus; [15, 16]), areas that correspond to those structures more affected in FTD. Additional studies in models of vascular dementia or alcohol-induced hippocampal damage have confirmed that the activation of CB_1_ receptors is important for the benefits obtained after FAAH inhibition in these pathological conditions [13, 46, 65, 81, 82]. The possible contribution of CB_1_ receptors to the effects of URB597 in our study is also suggested by our finding that the levels of this receptor in the mPFC are elevated in FTD mice at early ages (PND90) in the progression of the pathological phenotype. However, like anandamide, the other *N*-acylethanolamines (i.e. PEA and OEA), whose levels were shown to be elevated here in FTD mice, may also be elevated by FAAH inhibition, and produce neuroprotective actions by activating brain PPAR-α receptors (as in the case of OEA and PEA), or by desensitizing TRPV_1_ (as in the case of OEA and anandamide) and GPR55 (as in the case of PEA) receptors [14, 34]. FAAH also hydrolyzes other *N*-acyl-derivatives, such as *N*-acyl-taurines and some *N*-acyl-amino acids, which can exert neuroprotective actions via a variety of molecular targets [60, 61]. Therefore, the final answer to the question of which receptor mediates the protective effects of URB597 will require further ad hoc studies involving several different receptors. The same applies to the effects found with this inhibitor on glial cells, for example the particular resistance of GFAP-labelled cells in the hippocampal CA1 subfield compared to the benefits found after URB597 treatment in other CNS structures as the mPFC. As pointed out before, such situation may be related to the metabolism of anandamide (elevated by URB597 treatment) by COX-2 in these cells to generate prostamides, which, contrary to most endogenous FAAH substrates, may promote astrogliosis and result in neurotoxic effects exerted by mechanisms independent of cannabinoid receptors [37]. Indeed, previous studies have shown that FAAH inhibition can lead to the production of prostamides [45, 83] and induce pain and worsen inflammation rather than alleviating them [27, 45].

## Conclusions

In summary, our data confirmed that the FTD murine model used in this study recapitulates adequately the major neuropathological characteristics of this disease in humans, including behavioural and histopathological abnormalities also visible at later ages. We also showed that inhibiting FAAH with the subsequent potential elevation of the brain levels of endogenous endocannabinoids and endocannabinoid-like mediators (which together constitute the “endocannabinoidome”; [14]), and hence of the activity of their several neuroprotective and anti-inflammatory mediators, may represent a novel disease modifying therapy against TDP-43-induced neuropathology in FTD, serving to limit glial reactivity, preserve neuronal integrity and improve cognitive deficits. Further studies will try to confirm and move these promising results from their preclinical condition towards the clinical scenario.

## Supplementary Information

**Additional file 1: Figure S1**. Representative blots for the analysis of TDP-43 in the mPFC and the hippocampus of FTD and wildtype male mice at PND90, whose results are presented in Table 1.

**Additional file 2: Figure S2**. Behavioural data obtained in the Novel Object Recognition test during the training phase in FTD and wildtype male mice at PND90, whose details are described in the text. Data were expressed as means ± SEM and were analysed by the two-way analysis of variance followed by the Bonferroni test or the unpaired Student’s t-test.

**Additional file 3: Figure S3**. Behavioural data obtained in FTD and wildtype male mice at two different ages in a computer-aided actimeter, whose details are described in the text. Data were expressed as means ± SEM and were analysed by the unpaired Student’s t-test.

**Additional file 4: Figure S4**. Correlation analysis between the discrimination index, measured in the NOR test, and the T2 intensity or MT percentage, obtained by MRI analysis, in the cerebral cortex and the hippocampus of FTD and wildtype male mice at PND90. The methods used are described in the text. Data were assessed by a linear regression analysis to calculate the Pearson’s correlation coefficient.

**Additional file 5: Figure S5**. mRNA levels of several glia-related markers in the mPFC and the hippocampus of FTD and wildtype male mice at two different ages. The methods used are described in the text. Data were expressed as means ± SEM and were analysed by the unpaired Student’s t-test.

**Additional file 6: Figure S6**. Sox-2, GFAP and Ki67 immunofluorescence in the hippocampal dentate gyrus in FTD and wildtype male mice at PND90, including representative microphotographs for each genotype. The methods used are described in the text. Data were expressed as means ± SEM and were analysed by the unpaired Student’s t-test.

**Additional file 7: Figure S7**. Analysis of several proteins related to protein degradation in the mPFC and the hippocampus of FTD and wildtype male mice at PND90. The methods used are described in the text. Data were expressed as means ± SEM and were analysed by the unpaired Student’s t-test.

**Additional file 8: Figure S8**. Nissl staining and GFAP and Iba-1 immunofluorescence in the ventral horn of the spinal cord of FTD and wildtype male mice at PND365, including representative microphotographs for each genotype. The methods used are described in the text. Data were expressed as means ± SEM and were analysed by the unpaired Student’s t-test.

**Additional file 9: Figure S9**. Preference index measured in the NOR test and NeuN, Iba-1 and GFAP immunofluorescence in the hippocampal CA1 subfield of wildtype male mice after a chronic treatment with URB597 or vehicle, including representative microphotographs for each treatment. The methods used are described in the text. Data were expressed as means ± SEM and were analysed by the unpaired Student’s t-test.

## Data Availability

Data supporting reported results may be supplied upon request by authors.

## Abbreviations

2-AG: 2-Arachidonoyl glycerol
ADC: Apparent diffusion coefficient
ALS: Amyotrophic lateral sclerosis
Arg-1: Arginase-1
BSA: Bovine serum albumin
CB_1_: Cannabinoid receptor type-1
CB_2_: Cannabinoid receptor type-2
DAGL: Diacylglycerol lipase
EAAT2: Excitatory amino acid transporter-2
FAAH: Fatty acid amide hydrolase
FTD: Frontotemporal dementia
FTLD: Frontotemporal lobar degeneration
GWAS: Genome-wide association studies
IL-1β: Interleukin-1β
LC-APCI–MS: Liquid chromatography/atmospheric pressure chemical ionisation/mass spectrometry
MAGL: Monoacylglycerol lipase
mPFC: Medial prefrontal cortex
MRI: Magnetic resonance imaging
MT: Magnetization transfer
NAPE-PLD: N-arachidonoyl-phosphatidylethanolamine phospholipase D
NOR: Novel object recognition
OEA: Oleylethanolamide
PEA: Palmitoylethanolamide
PND90: Postnatal day 90
TNF-α: Tumour necrosis factor-α
