# Supplementary figures and images for: Preclinical investigation in FAAH inhibition as a neuroprotective therapy for frontotemporal dementia using TDP-43 transgenic male mice
Source: J Neuroinflammation. 2023 May 6;20:108. doi: 10.1186/s12974-023-02792-z (PMC10163746; doi:10.1186/s12974-023-02792-z)

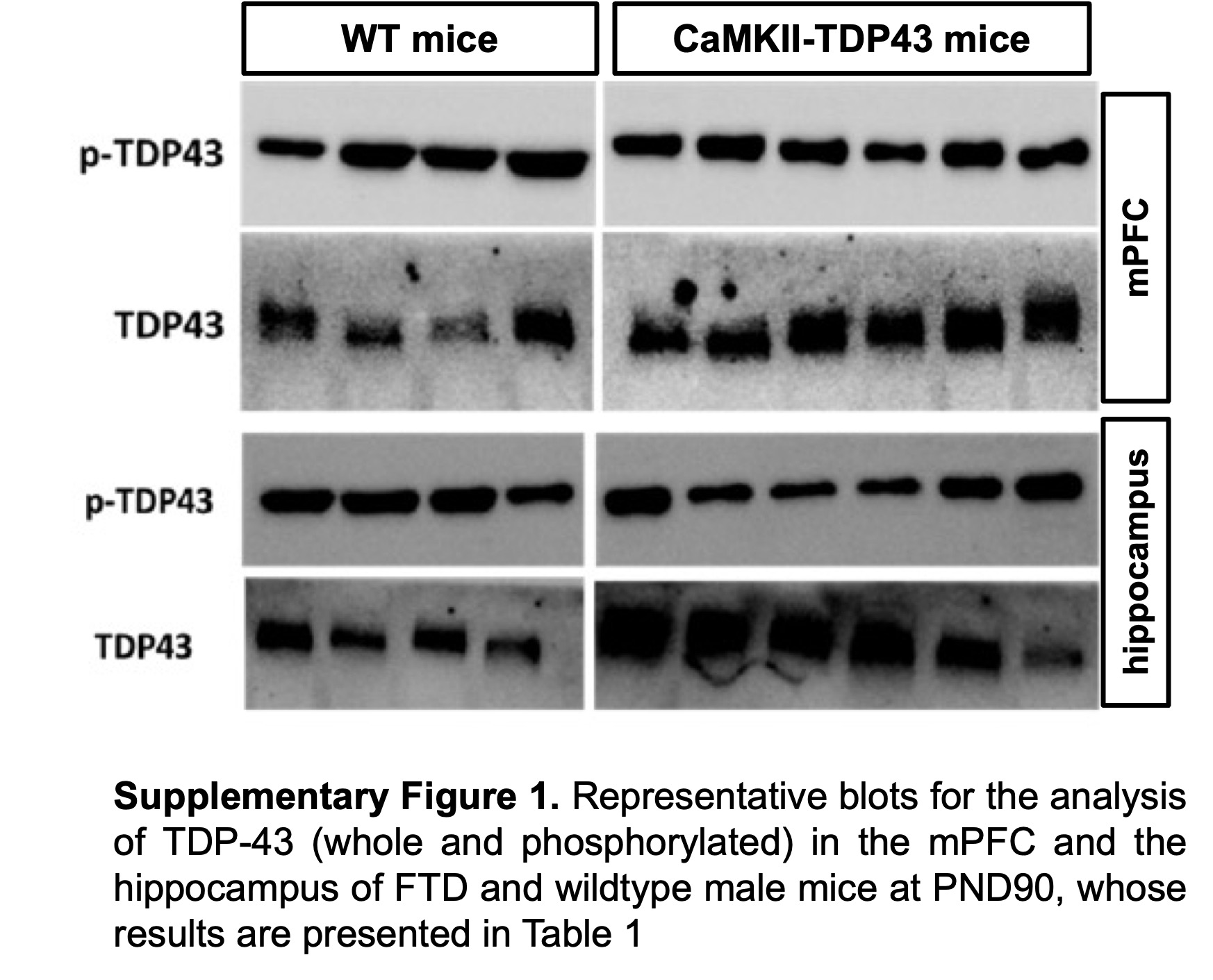

Supplement: Supplementary file 1 — Additional file 1: Figure S1. Representative blots for the analysis of TDP-43 in the mPFC and the hippocampus of FTD and wildtype male mice at PND90, whose results are presented in Table 1. [file 12974_2023_2792_MOESM1_ESM.tif]

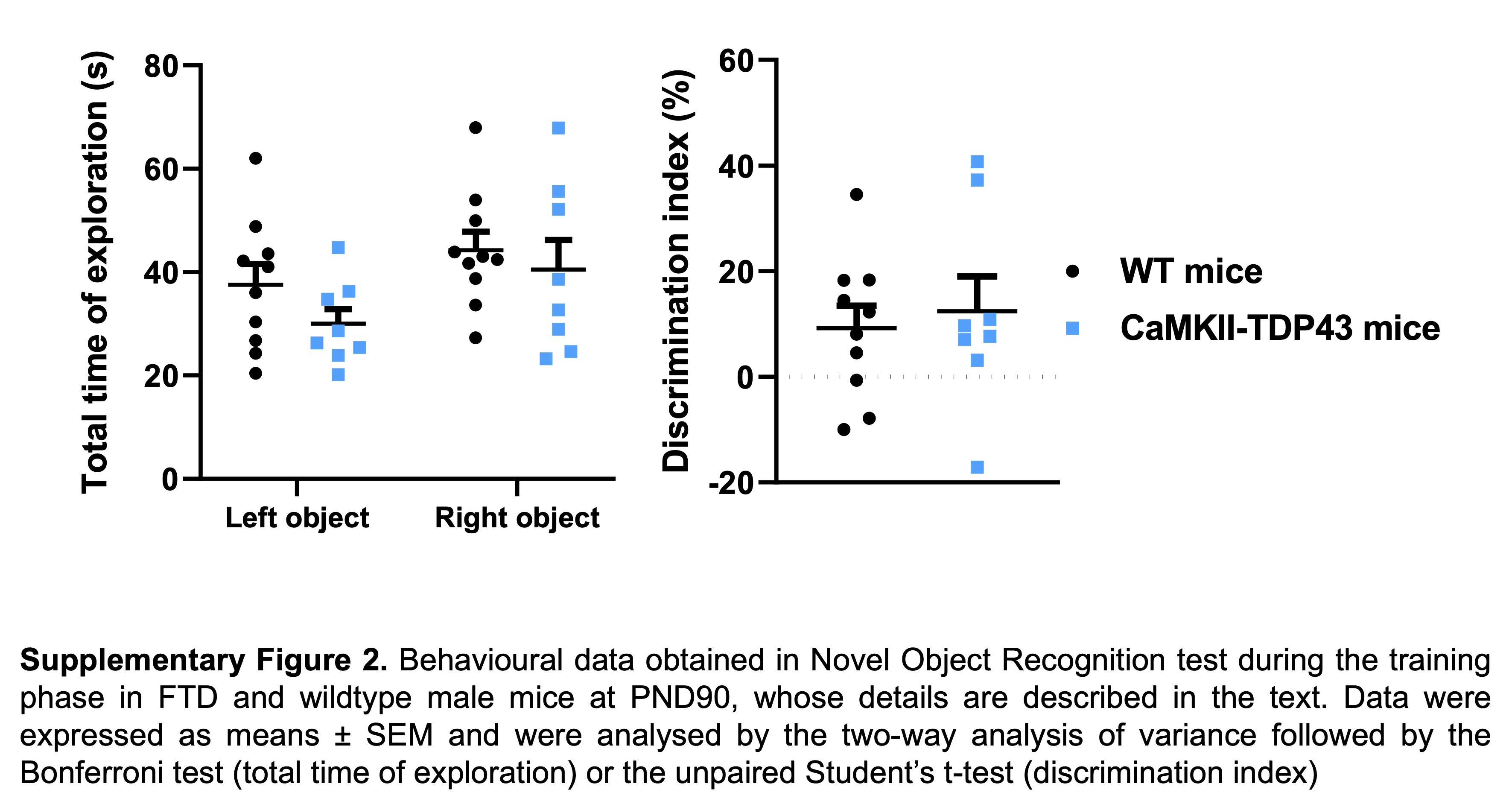

Supplement: Supplementary file 2 — Additional file 2: Figure S2. Behavioural data obtained in the Novel Object Recognition test during the training phase in FTD and wildtype male mice at PND90, whose details are described in the text. Data were expressed as means ± SEM and were analysed by the two-way analysis of variance followed by the Bonferroni test or the unpaired Student’s t-test. [file 12974_2023_2792_MOESM2_ESM.tif]

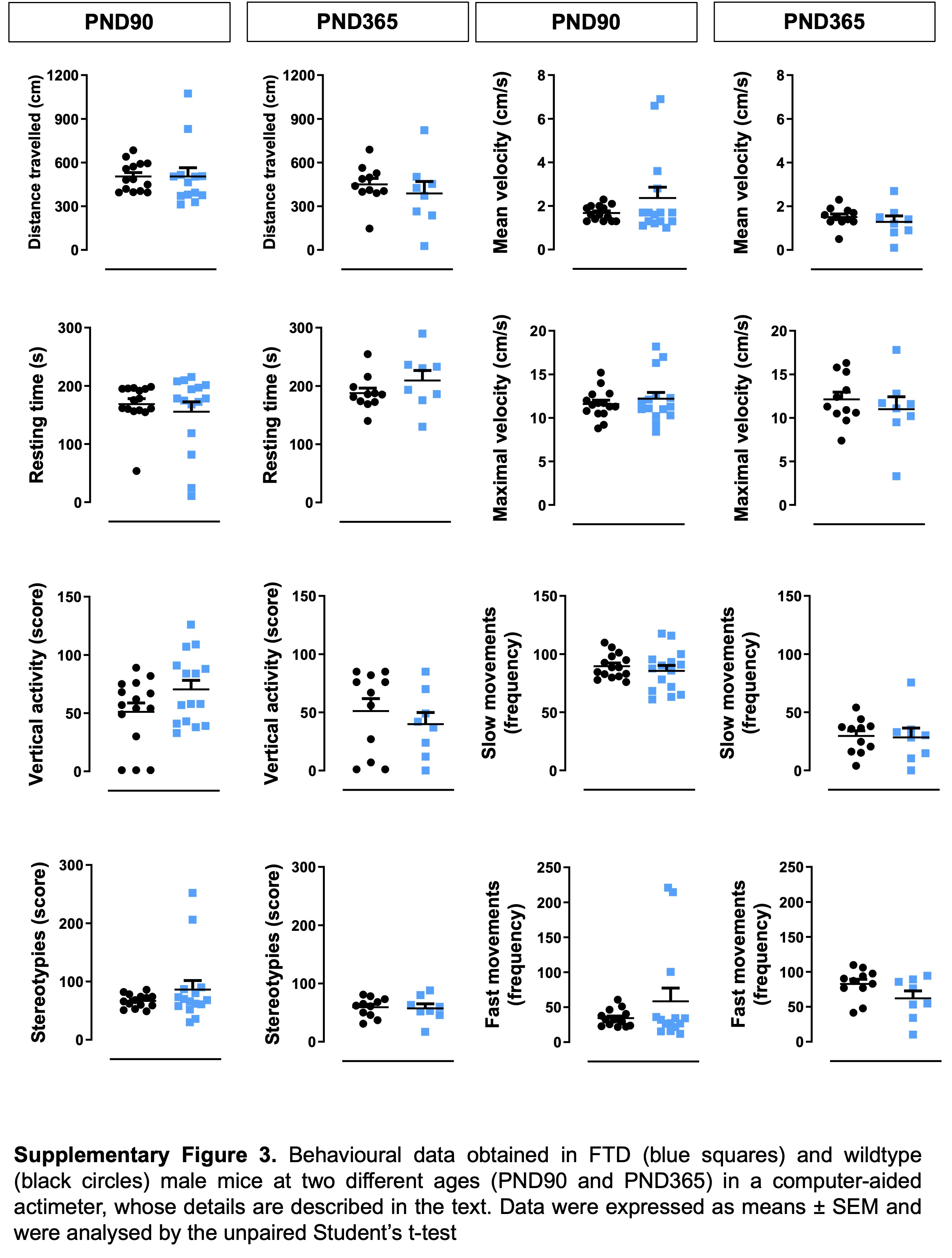

Supplement: Supplementary file 3 — Additional file 3: Figure S3. Behavioural data obtained in FTD and wildtype male mice at two different ages in a computer-aided actimeter, whose details are described in the text. Data were expressed as means ± SEM and were analysed by the unpaired Student’s t-test. [file 12974_2023_2792_MOESM3_ESM.tif]

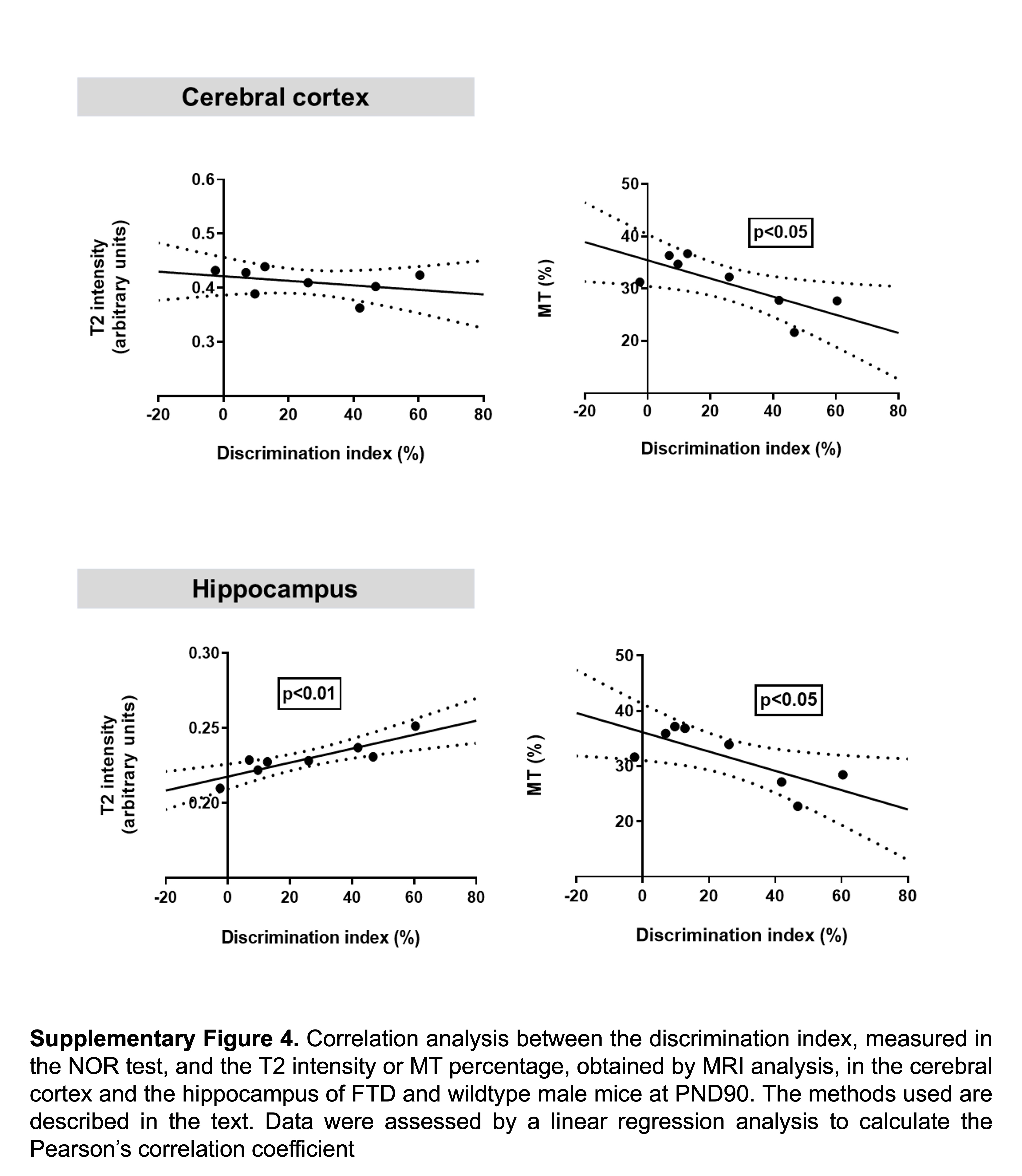

Supplement: Supplementary file 4 — Additional file 4: Figure S4. Correlation analysis between the discrimination index, measured in the NOR test, and the T2 intensity or MT percentage, obtained by MRI analysis, in the cerebral cortex and the hippocampus of FTD and wildtype male mice at PND90. The methods used are described in the text. Data were assessed by a linear regression analysis to calculate the Pearson’s correlation coefficient. [file 12974_2023_2792_MOESM4_ESM.tif]

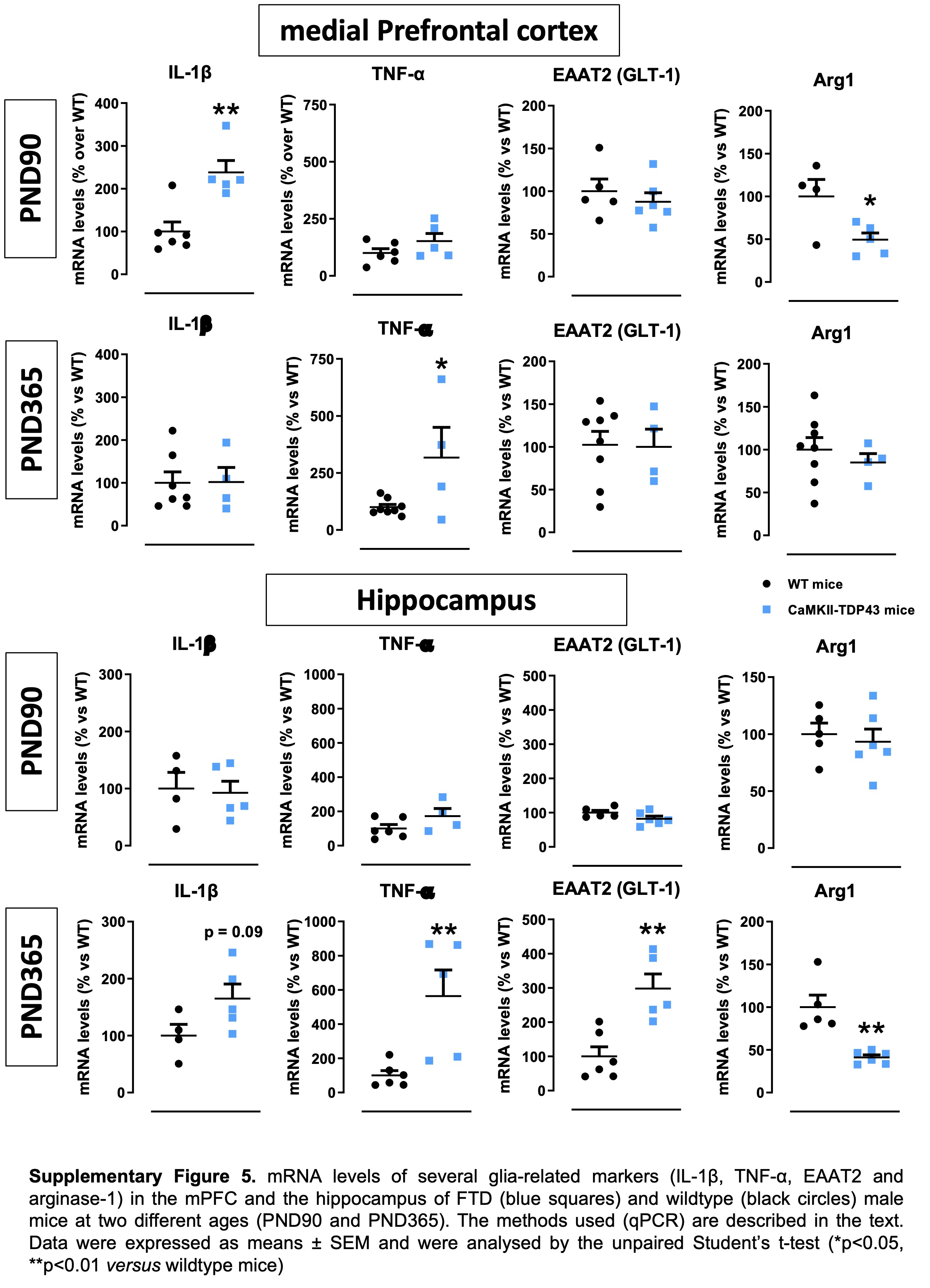

Supplement: Supplementary file 5 — Additional file 5: Figure S5. mRNA levels of several glia-related markers in the mPFC and the hippocampus of FTD and wildtype male mice at two different ages. The methods used are described in the text. Data were expressed as means ± SEM and were analysed by the unpaired Student’s t-test. [file 12974_2023_2792_MOESM5_ESM.tif]

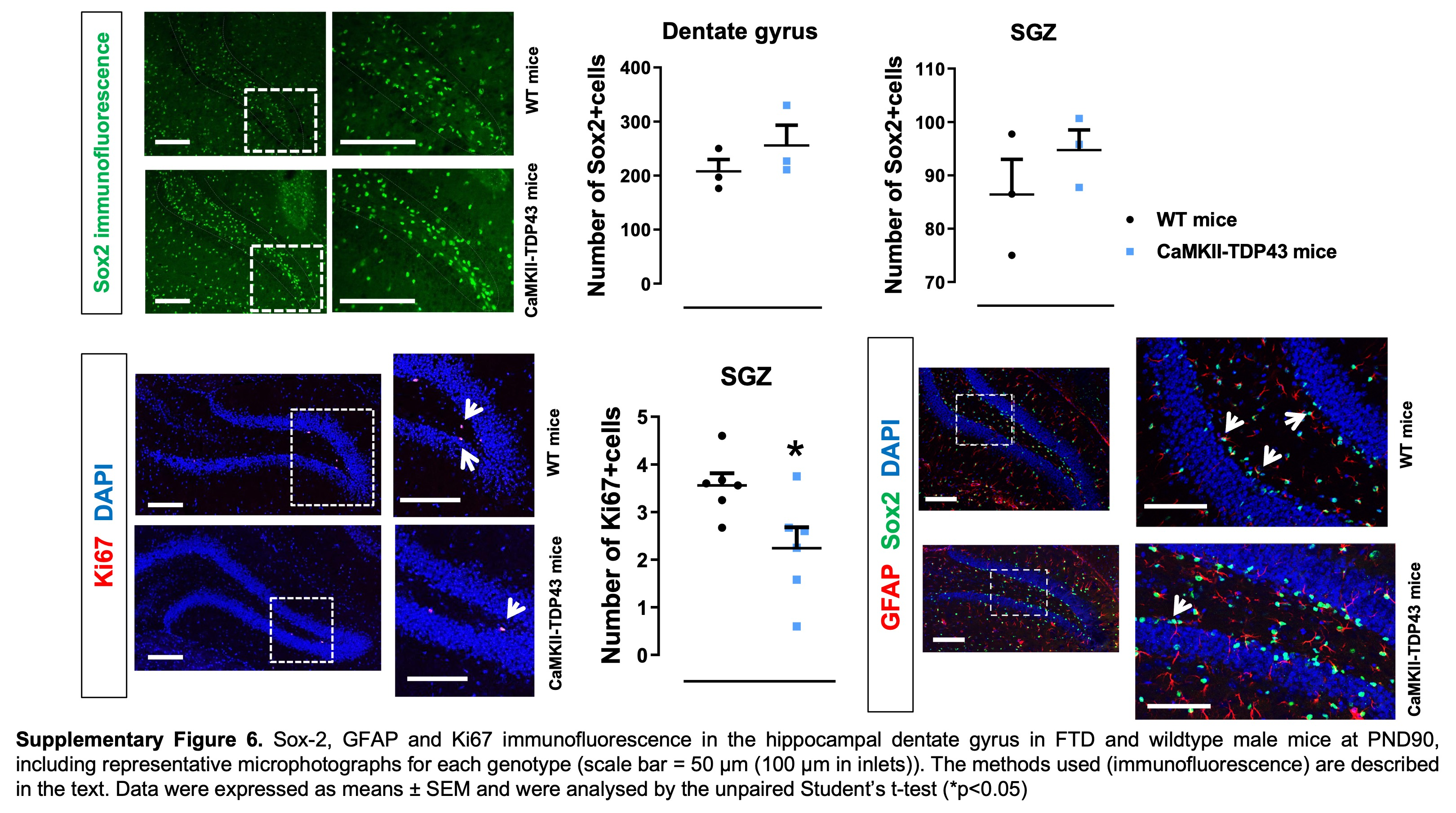

Supplement: Supplementary file 6 — Additional file 6: Figure S6. Sox-2, GFAP and Ki67 immunofluorescence in the hippocampal dentate gyrus in FTD and wildtype male mice at PND90, including representative microphotographs for each genotype. The methods used are described in the text. Data were expressed as means ± SEM and were analysed by the unpaired Student’s t-test. [file 12974_2023_2792_MOESM6_ESM.tif]

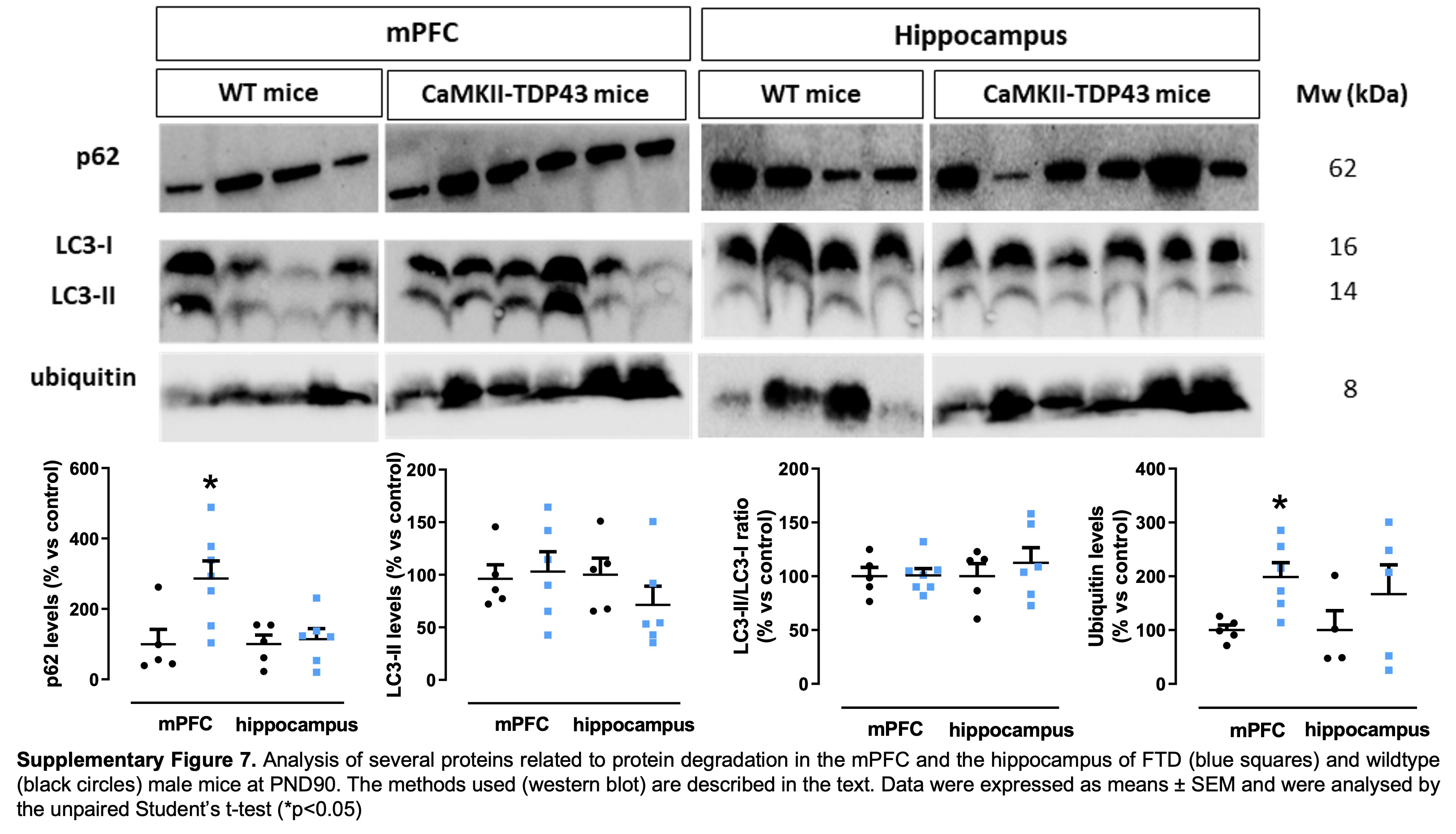

Supplement: Supplementary file 7 — Additional file 7: Figure S7. Analysis of several proteins related to protein degradation in the mPFC and the hippocampus of FTD and wildtype male mice at PND90. The methods used are described in the text. Data were expressed as means ± SEM and were analysed by the unpaired Student’s t-test. [file 12974_2023_2792_MOESM7_ESM.tif]

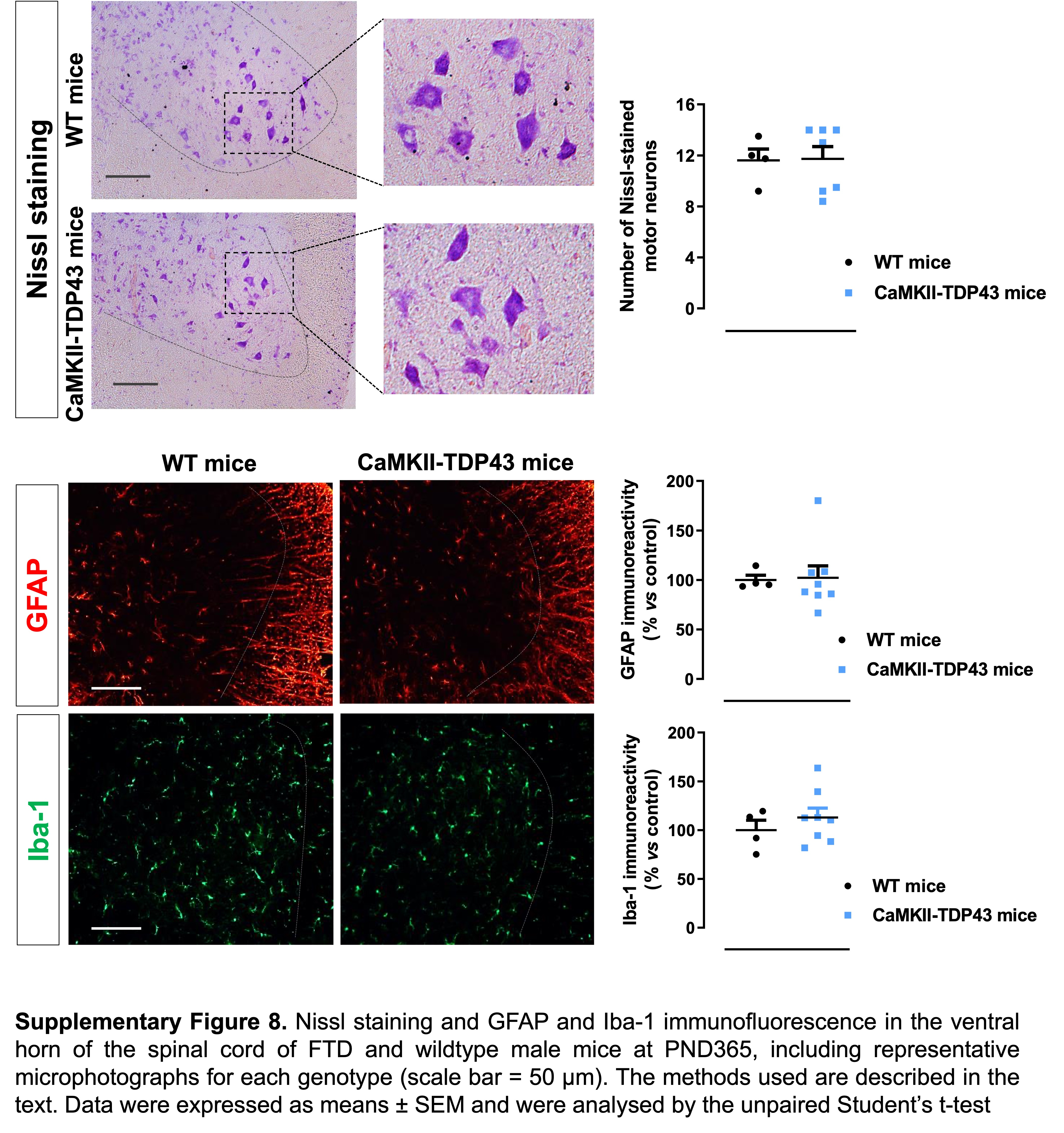

Supplement: Supplementary file 8 — Additional file 8: Figure S8. Nissl staining and GFAP and Iba-1 immunofluorescence in the ventral horn of the spinal cord of FTD and wildtype male mice at PND365, including representative microphotographs for each genotype. The methods used are described in the text. Data were expressed as means ± SEM and were analysed by the unpaired Student’s t-test. [file 12974_2023_2792_MOESM8_ESM.tif]

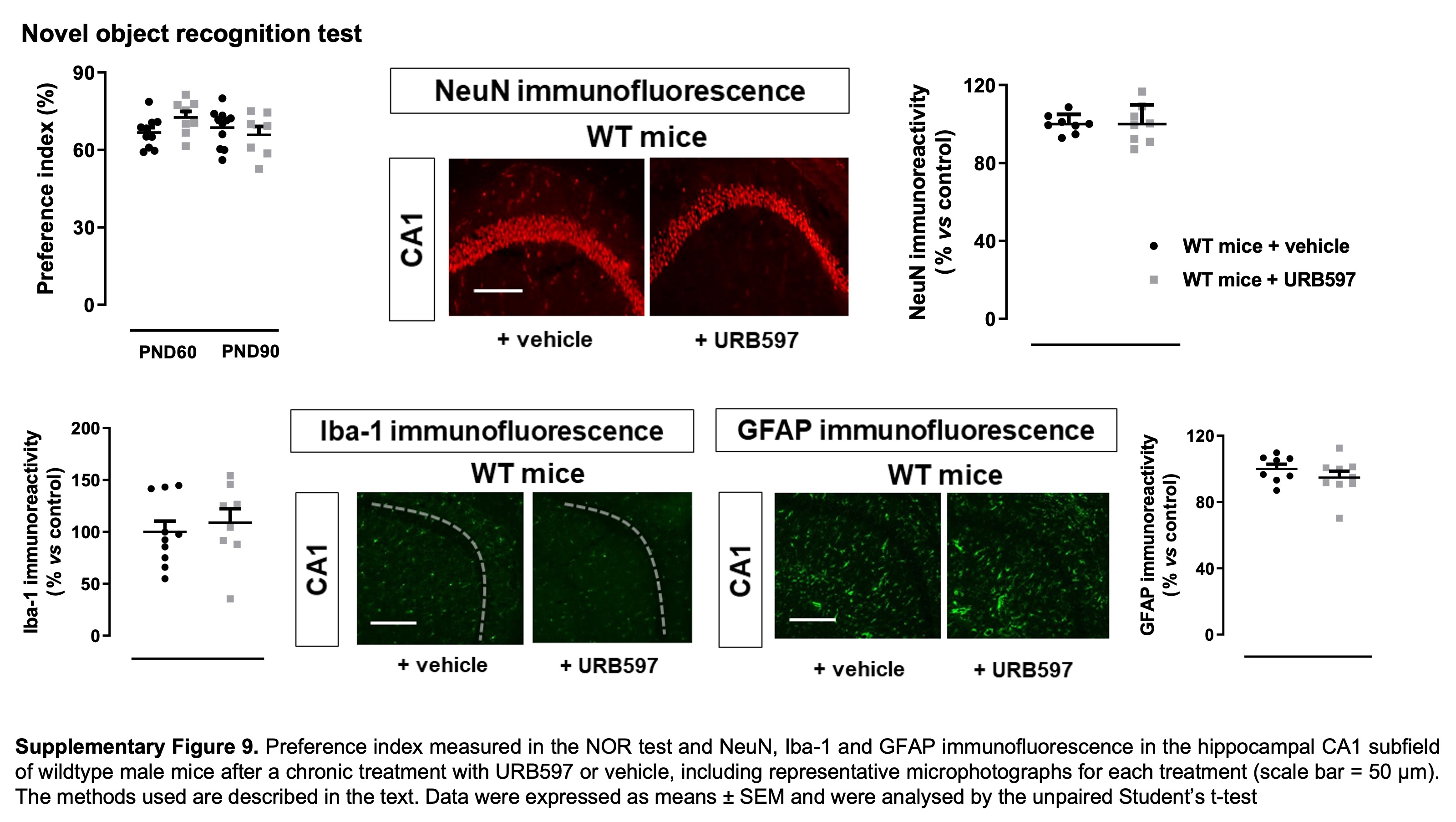

Supplement: Supplementary file 9 — Additional file 9: Figure S9. Preference index measured in the NOR test and NeuN, Iba-1 and GFAP immunofluorescence in the hippocampal CA1 subfield of wildtype male mice after a chronic treatment with URB597 or vehicle, including representative microphotographs for each treatment. The methods used are described in the text. Data were expressed as means ± SEM and were analysed by the unpaired Student’s t-test. [file 12974_2023_2792_MOESM9_ESM.tif]
